# Psychophysiological Effects of Downregulating Negative Emotions: Insights From a Meta-Analysis of Healthy Adults

**DOI:** 10.3389/fpsyg.2020.00470

**Published:** 2020-04-16

**Authors:** Jenny Zaehringer, Christine Jennen-Steinmetz, Christian Schmahl, Gabriele Ende, Christian Paret

**Affiliations:** ^1^Department of Psychosomatic Medicine and Psychotherapy, Central Institute of Mental Health Mannheim, Medical Faculty Mannheim, Heidelberg University, Mannheim, Germany; ^2^Department Neuroimaging, Central Institute of Mental Health Mannheim, Medical Faculty Mannheim, Heidelberg University, Mannheim, Germany; ^3^Department of Biostatistics, Central Institute of Mental Health Mannheim, Medical Faculty Mannheim, Heidelberg University, Mannheim, Germany; ^4^Department of Psychiatry, Schulich School of Medicine and Dentistry, Western University, London, ON, Canada; ^5^Sagol Brain Institute, Wohl Institute for Advanced Imaging, Tel-Aviv Sourasky Medical Centre, Tel-Aviv, Israel

**Keywords:** meta-analysis, emotion regulation, psychophysiology, reappraisal, suppression, autonomic nervous system, electromyography

## Abstract

Assessing psychophysiological responses of emotion regulation is a cost-efficient way to quantify emotion regulation and to complement subjective report that may be biased. Previous studies have revealed inconsistent results complicating a sound interpretation of these findings. In the present study, we summarized the existing literature through a systematic search of articles. Meta-analyses were used to evaluate effect sizes of instructed downregulation strategies on common autonomic (electrodermal, respiratory, cardiovascular, and pupillometric) and electromyographic (corrugator activity, emotion-modulated startle) measures. Moderator analyses were conducted, with moderators including study design, emotion induction, control instruction and trial duration. We identified *k* = 78 studies each contributing multiple sub-samples and performed 23 meta-analyses for combinations of emotion regulation strategy and psychophysiological measure. Overall, results showed that effects of reappraisal and suppression on autonomic measures were highly inconsistent across studies with rather small mean effect sizes. Electromyography (startle and corrugator activity) showed medium effect sizes that were consistent across studies. Our findings highlight the diversity as well as the low level of standardization and comparability of research in this area. Significant moderation of effects by study design, trial duration, and control condition emphasizes the need for better standardization of methods. In addition, the small mean effect sizes resulting from our analyses on autonomic measures should be interpreted with caution. Findings corroborate the importance of multi-channel approaches.

Emotion regulation is a vital part of our daily lives. It permits individuals to control the occurrence, intensity, type, and duration of emotions (Gross and Thompson, 2007). Strategies to regulate emotions not only alter the subjective experience of emotions (Gross, 1998a), but also map onto bodily responses such as changes in measures of the autonomic nervous system (Gross, 2002; Webb et al., 2012), emotion-expressive behavior (Dan-Glauser and Gross, 2011, 2015), somatic reflexes such as the emotion-modulated startle (Jackson et al., 2000), or neural activation (Ochsner et al., 2004; Buhle et al., 2014). The habitual use of adaptive emotion regulation strategies is a hallmark of successful functioning and is associated with increased well-being, whereas difficulties with regulating emotions have been linked to many psychopathologies (Aldao et al., 2010; Joormann and Vanderlind, 2014; Schmahl et al., 2014). In light of the significance of emotion regulation, appropriate experimental paradigms are required that are suitable for research involving large sample sizes and patient populations.

In a typical emotion regulation study, emotions are experimentally induced using affective stimuli such as films (Gross and Levenson, 1995) or pictures (e.g., International affective picture system; Lang et al., 2009). Participants are instructed to regulate their emotional experience or to respond naturally without regulating their emotions (i.e., the control condition). By comparing the regulation with the control condition it is possible to determine the effect of regulation, which has been used as an indirect measure of emotion regulation effectiveness (Webb et al., 2012).

Assessing psychophysiological correlates has several important advantages. They move beyond on-line self-reports and retrospective assessments, as physiological responding is regarded as automatic, relatively unconscious, and fast (Bradley et al., 1993b; Öhman and Soares, 1994; Edelmann and Baker, 2002; Olsson and Phelps, 2004; Lapate et al., 2014). Research focusing on the direct effects of emotion regulation has found significant psychophysiological changes even when subjective experience remained unaffected (Gross and Levenson, 1993, 1997). Hence, psychophysiological measures can offer important insights into internal emotional experiences that are not available by assessing self-report. In addition, psychophysiological responses are easier to assess than neural physiological measures (e.g., functional magnetic resonance imaging) and are thus cost-efficient methods for quantifying differences in emotion regulation.

## Conceptual Foundations of Emotion Regulation

There have been multiple attempts to classify emotion regulation strategies (Gross, 1998a,b; Larsen, 2000; Koole, 2009). One of the most influential models is the *process model of emotion regulation* (Gross, 1998a,b, 2015), which broadly categorizes strategies as either being antecedent-focused, i.e., strategies are implemented before the emotional response has fully unfolded, and as response-focused, i.e., strategies are implemented after the emotional response has already been generated. The process model distinguishes five major emotion regulation processes: situation selection (i.e., attempts to change a future emotional response), situation modification (i.e., changing the situation in order to modify its emotional effect), attentional deployment (i.e., distraction away from or concentration on an emotional stimulus to modify the emotion itself), cognitive change (i.e., reappraise a situation or to change the perspective so that the emotional experience is modulated), and response modulation (i.e., strategies to suppress expressive behavior, thoughts, or emotions). Situation selection, situation modification, attentional deployment, and cognitive change are regarded as antecedent-focused and response modulation is regarded as a response-focused process.

A majority of past emotion regulation studies have instructed participants to distract themselves from, reappraise or suppress^1^ a target stimulus in order to downregulate emotions. These strategies correspond to attentional deployment, cognitive change, and response modulation, respectively, In addition, a considerable number of studies allowed participants to use a strategy of their own choice (Jackson et al., 2000; Dillon and LaBar, 2005; Piper and Curtin, 2006; Lissek et al., 2007; Driscoll et al., 2009; Lee et al., 2009; Golkar et al., 2014; Baur et al., 2015; Conzelmann et al., 2015; Grillon et al., 2015). The present meta-analysis thus focuses on these four major types of downregulation instructions, that is distraction, reappraisal, suppression, and downregulation instructions that allowed participants to choose their own strategy. Other strategies were out of the scope. For a comprehensive overview see Table 1.

**Table 1:** Emotion downregulation processes and their strategies considered in this meta-analysis.

**Process**	**Strategy**	**Subtype**	**Example**
**EMOTION REGULATION INSTRUCTIONS**			
Attentional deployment	Distraction	Active distraction	Participants are instructed to think about something positive or neutral that is unrelated to the target emotion/stimulus
Cognitive Change	Reappraisal	Reinterpret the emotional stimulus	Participants are instructed to reinterpret the emotional stimulus to decrease the target emotion
		Reappraise via perspective taking, i.e., distancing	participants are instructed to alter the impact of a stimulus by adopting a more objective perspective
		Reappraise Mixed	A mixture of reappraisal instructions
Response modulation	Suppression	Suppress the expression of emotion	Participants are instructed to hide the way they are feeling, e.g., not to smile
		Suppress the experience of an emotion	Participants are instructed to suppress their emotional experience
		Suppress thoughts of the emotion eliciting event	Participant are instructed to suppress thoughts about the emotion-eliciting event
		Suppression mixed	A mixture of suppression instructions
Downregulation unspecified	Own choice	Own choice	Participants are free to choose a strategy that works best for them. They are not allowed to create a different emotion or think of something unrelated to the stimulus
**CONTROL INSTRUCTIONS**			
		No instruction (C1)	No instructions are given
		Instructions not to regulate (C2)	Participants are told that they should not use a regulation strategy
		Instructions to maintain (C3)	Participants are instructed to maintain the target emotion
		Instructions to experience naturally (C4)	Participants are instructed to respond naturally without regulating it
		Control mixed (C5)	A mixture of control instructions

## Psychophysiological Responses of Emotions and Emotion Regulation

There is great interest in understanding the relationship between emotions and psychophysiological responses including responses of the autonomic nervous system (i.e., cardiovascular, electrodermal, respiratory, pupillometric) and responses measured with the electromyogram (EMG) such as facial muscle activity (e.g., corrugator supercilii activity) and somatic reflexes (e.g., emotion-modulated startle). The interested reader is directed to detailed reviews by Cacioppo et al. (2000), Kreibig (2010), Siegel et al. (2018), and Stemmler (2004). See Table 2 for an overview of relevant psychophysiological measures within the emotion regulation literature. Such relations have most commonly been studied in terms of two affective dimensions, that is valence (positive-negative) and arousal (high-low) (Lang, 1995; Bradley et al., 2001). Some measures such as heart rate, emotion-modulated startle, and facial activity are specific to the valence of the emotion (Bradley et al., 2001) and others such as skin conductance and pupil dilation are more specific to the arousal dimension (Greenwald et al., 1989; VanOyen Witvliet and Vrana, 1995; Bradley et al., 2001). Past research has also put a lot of effort into answering the question whether different emotion categories (e.g., disgust, sadness, fear) produce distinct physiological response patterns. In a recent meta-analysis the hypothesis could not be confirmed (Siegel et al., 2018). Rather, emotions seem to elicit an unspecific set of psychophysiological changes.

**Table 2:** Common psychophysiological measures of emotion regulation studies.

**Body system**	**Measurement**	**Abbreviation**	**Measurement system (units)**	**Description**
Cardiovascular	Cardiac output	CO	l/min	Blood volume pumped by the heart per minute.
	**Diastolic blood pressure**	**DBP**	**mmHg**	Lowest blood pressure of circulating blood on the walls of blood vessels in between two heartbeats, measured in millimeters of mercury.
	**Ear pulse transit time**	**EPTT**	**ms**	Time interval between the R-wave of the electrocardiogram to the pulse wave arrival at the ear.
	**Finger pulse amplitude**	**FPA**	**Arbitrary**	Amplitude of the pulse waveform measured in the finger. Indicator of dilation and constriction of the blood vessels.
	**Finger pulse transit time**	**FPTT**	**ms**	Time interval between the R-wave of the electrocardiogram to the pulse wave arrival at the finger.
	**Heart rate/interbeat interval/heart period**	**HR/HP**	**bpm/ms/ms**	Number of beats per unit of time/time between heart beats (inverse of heart rate).
	**Heart rate variability**	**HRV**	**Units vary by method**	Variation in heart rate. Refers specifically to the high-frequency HRV [also called respiratory sinus arrhythmia (RSA)].
	Low frequency HRV	LF	Units vary by method	Variation in heart rate. Refers specifically to the low-frequency HRV.
	Ratio of low- and high-frequency HRV	LF/HF	Units vary by method	Variation in heart rate. Refers specifically to the ratio between low- and high-frequency HRV.
	**Mean arterial pressure**	**MAP**	**mmHg**	Mean blood pressure of circulating blood on the walls of blood vessels in between two heartbeats, measured in millimeters of mercury.
	Pre-ejection period	PEP	ms	Period between the beginning of electrical stimulation of the heart to the opening of the aortic valve. Indicator of the cardiac contractile force (i.e., how hard the heart is beating).
	Stroke volume	SV	mL	Volume of blood pumped from the left ventricle per beat.
	**Systolic blood pressure**	**SBP**	**mmHg**	Maximum blood pressure of circulating blood on the walls of blood vessels in between two heartbeats, measured in millimeters of mercury.
	Total peripheral resistance	TPR	Unity vary by method	Overall resistance that must be overcome to push blood through the whole circulatory system (i.e., all major arterial trees).
Electrodermal	**Skin**, **conductance response**	**SCR**	**MicroSiemens**	Peak amplitude, magnitude or local maximum of the skin conductance response. Includes non-specific skin conductance responses during longer periods of time if reported as amplitude.
	**Skin conductance level**	**SCL**	**MicroSiemens**	Mean change of skin conductance over a specific period of time. Operationalized as simple average, change from baseline, area under the curve or integrated signal.
	Number of skin conductance responses	nSCR	n	Number of skin responses per unit of time (e.g. per minute).
Respiratory	Inspiration/expiration time	IT/ET	sec	Average inhalation/exhalation time per respiratory cycle.
	**Respiration amplitude**	**RA**	**mL**	Difference in volts between the point of maximum inspiration and the point of maximum expiration.
	Respiration rate	RR	c/min	Number of breaths per minute.
	Tidal volume	TV	mL	Air volume that moves into or out of the lungs while breathing quietly.
Pupillometric	**Pupil dilation**	**PD**	**mm**	Average diameter of pupil in millimeter during a specific period of time.
Electromyographic	**Emotion-modulated startle**	**Startle**	**MicroVolt**	Amplitude of the startle eyeblink response (orbicularis oculi) in response to affective stimuli.
	**Corrugator supercilii activity**	**cEMG**	**MicroVolt**	Muscular activity of the corrugator supercilii responsible for furrowing of the brow.
	Zygomaticus major activity	zEMG	MicroVolt	Muscular activity of the zygomaticus major responsible for smiling.
Other	**Finger temperature**	**FT**	**F/C°**	Temperature of the finger, in Fahrenheidt (F) or Celcius (C°).

*The measures in bold were included in our meta-analysis; for the other measures the number of studies was insufficient (k < 5 studies per cell). Because heart rate (HR) and interbeat interval (IBI) are inversely related, we switched the direction of the effect sizes when IBI was extracted (instead of HR). Descriptions derived and adapted from Berntson et al. (2016), Blumenthal et al. (2005), Cacioppo et al. (2000), Dawson et al. (2016), and Siegel et al. (2018)*.

When it comes to the *regulation* of emotions, much evidence has accumulated suggesting that suppression is related to an increase in sympathetic nervous system activity but no difference in self-report to negative stimuli (Gross and Levenson, 1993, 1997; Richards and Gross, 1999). The enhanced sympathetic activation following suppression has led researchers to conclude that suppression “exacts a palpable physiological cost” (Gross and Levenson, 1997, p. 101). In other words, because response-focused strategies involve an active modulation of expressive behavior, increased sympathetic activation might be the result of that effort (Butler et al., 2003). In contrast, past literature has proposed that reappraisal has little impact on sympathetic and cardiovascular measures (Gross, 1998a). A meta-analysis studying the overall physiological effect of different emotion regulation strategies confirmed this general pattern: cognitive change had a smaller effect on physiology than response modulation (Webb et al., 2012).

However, as noted earlier, there is a vast range of different psychophysiological outcome measures ranging from cardiovascular, electrodermal, respiratory, pupillometric, and electromyographic response systems and it has been shown that the nature of the relationship between cognitive emotion regulation and different psychophysiological responses can vary largely (Bernat et al., 2011). By simply combining all psychophysiological measures to a composite score is helpful in looking at the overall effectiveness of an emotion regulation strategy (as has been done in the meta-analysis by Webb et al., 2012), but it does not reveal which of the individual psychophysiological responses change or do not change with an emotion regulation strategy.

When looking at individual psychophysiological measures, findings are mixed with respect to the effects of emotion regulation on autonomic physiology. Reappraisal instructions focusing on decreasing negative emotions compared to a control condition have been shown to have no effect on (Gross, 1998a; Kalisch et al., 2005; Goldin et al., 2019), increase (Sheppes et al., 2009; Lohani and Isaacowitz, 2014), or decrease (Urry et al., 2009) skin conductance and to increase (Urry et al., 2006; van Reekum et al., 2007) or decrease (Bebko et al., 2011) pupil diameter. Contradictory patterns can also be found for suppression strategies. For example, individuals' heart rate was significantly increased (Hagemann et al., 2006; Ben-Naim et al., 2013), decreased (Gross and Levenson, 1993; Robinson and Demaree, 2009), or stayed the same (Gross, 1998a) when individuals suppressed negative emotions compared to a control condition. These inconsistencies may be due to the large heterogeneity between studies, which can substantially affect the magnitude of the physiological responses. The contradictory pattern of results across the literature does not allow a straightforward interpretation. The causes for these inconsistencies are, however, not well-understood, and this inevitably obscures the detection of common trends.

## Factors Related to the Impact of Emotion Regulation on Psychophysiology

### Study Design

Studies using within-study designs found larger effects of emotion regulation on experiential, behavioral and physiological outcomes than did studies employing between-study designs (cf. Webb et al., 2012). Employing within-study designs reduces sampling error thereby increasing power. On the other hand, within-study designs may also increase task difficulty because participants are required to engage in more than just one emotion regulation strategy. In event-related designs typical for within-subject studies, participants may even shift continuously between different strategies.

### Emotion Induction

Emotion regulation studies have used a variety of different emotional stimuli, including pictures (e.g., the International Affective Picture System; IAPS: Lang et al., 2009), film clips (Gross and Levenson, 1995), stressful tasks (e.g., the Trier Social Stress Test; Kirschbaum et al., 1993), dyadic interactions (Levenson and Gottman, 1983), or threat of shock paradigms (Delgado et al., 2008). Each type of stimulus provides a reliable method to generate emotions. However, a key dimension on which induction methods differ is whether they require participants to sit passively in front of a monitor or whether they employ a stressful task or conversation with a (romantic) partner. Somatic activity has a significant influence on autonomic response measures, especially on heart rate (Obrist, 1981). In addition, stressful tasks such as giving a speech alter the sympathetic nervous system to a stronger degree than picture viewing (Fechir et al., 2008). When it comes to potential differences between films and pictures, findings are mixed. Studies on emotion processing have been shown that e.g., heart rate returns to baseline if the picture remains still, but further slows down if the picture involves motion (Detenber et al., 1998; Simons et al., 1999). However, a recent study on emotion regulation reported that films and pictures did not differently affect the emotion regulation process on a physiological level, although films elicited a stronger absolute skin conductance response than pictures (Morawetz et al., 2016a). We are not aware of any other study directly assessing the impact of the emotion induction method on psychophysiological effects in the context of emotion regulation and thus we will address this question in the present analysis^2^.

### Control Instruction

Effects of emotion regulation strategies on psychophysiological measures can be determined by contrasting the emotion regulation instruction against different control instructions. For example, participants can be instructed to “maintain” the emotion they feel (Jackson et al., 2000), to “view” the emotional stimulus (Gross and Levenson, 1993), or to “respond naturally” (Shiota and Levenson, 2009). Previous literature has shown that differences in neural activation depend on the control condition instruction (Schaefer et al., 2002), with higher amygdala activation reported for “maintain” than for “view” instructions. The terminology used as control instructions (e.g., maintain vs. view) has not been systematically explored in psychophysiological studies of emotion regulation yet. However, it could have important influences on physiological processes as shown by an fMRI study (Diers et al., 2014). Similarly, Webb et al. (2012) found that the control condition moderated the physiological effects of emotion regulation (Webb et al., 2012).

### Trial Duration

Another important aspect of the study design which varies largely across studies is the trial duration of the regulation period. According to the implementation and maintenance model (Kalisch, 2009; Paret et al., 2011), reappraisal for example is divided into two phases: In the early phase, participants choose and implement a regulation strategy, whereas in the late phase they maintain the strategy in working memory and monitor its success. Hence, reappraisal might need several seconds until it effectively reduces negative emotions. Thus, the effect of reappraisal might become larger with increasing trial duration, which might also affect physiology.

## Aim of Study

The primary aim of the present study was to quantitatively summarize the relation between popular emotion downregulation instructions (distraction, reappraisal, suppression, own choice) and common psychophysiological measures (i.e., cardiovascular, electrodermal, respiratory, pupillometric, electromyographic) in healthy adults. In light of the contradictory pattern of psychophysiological effects in the emotion regulation literature we aimed to answer the following questions: (a) What are the effects of distraction, reappraisal, suppression, and downregulation where participants choose a strategy that works best for them on individual psychophysiological response measures? (b) How consistent are these effects across studies? and (c) What aspects of the study design moderate the effects? In light of the hypothesis that psychophysiological measures are somewhat sensitive to the valence of the induced emotion and because the majority of studies on emotion regulation and psychophysiology induced negative emotions, the present meta-analysis focuses on the downregulation of negative stimuli (for an overview of studies employing positive stimuli see Table S1).

We first systematically searched for emotion regulation studies that instructed participants to use emotion regulation strategies and that assessed psychophysiological measures of our interest as dependent variable. To advance current knowledge, we performed meta-analyses to separately quantify the effects for each of these measures during emotion regulation. In addition, we performed moderator analyses to explore the impact of study characteristics on the effect sizes. Moderators of interest were study design, trial duration, control instruction, and emotion induction method. It is important to note that our ability to identify the effects of cognitive emotion regulation strategies on psychophysiological variables and potential moderators is limited by the published studies available for meta-analysis.

## Methods

### Selection of Studies

Studies were identified through a systematic literature search of articles using the PubMed, Web of Science, and PsychINFO databases. The search strategy was developed to maximize the sensitivity of article identification by combining individual words and medical subject headings (MeSH)^1^. We searched for the keywords *emotion regulation or emotional regulation* cross referenced with *psychophysiology* [MeSH], psychophysiologic^*^, autonomic, parasympathetic, sympathetic, respiration [MeSH], *cardiovascular, electrocardiography* [MeSH]*, respiratory sinus arrhythmia* [MeSH], *blood pressure* [MeSH]*, heart rate* [MeSH], *startle, startle reflex* [MeSH], *electromyography* [MeSH], *pupil diameter, pupil dilation, electrodermal or skin conductance*, and *galvanic skin response* [MeSH] cross referenced with stimulus, stimuli, film^*^, picture^*^, image^*^, script^*^, anxiety, fear^*^, threat^*^, and video^*^. Additionally, reference lists from identified studies that met the inclusion criteria (see the next section for criteria) as well as relevant articles in the authors' library were reviewed for titles that might have been previously missed. Subsequently, studies identified in this manner (*n* = 13) were collected for inclusion.

The search process described above yielded a total of 1,353 potentially relevant articles on July 18, 2019 (after duplicates were removed)^3^. The first author and another independent reviewer (Stephanie Mall, research assistant) systematically examined titles and relevant abstracts using the Covidence website (www.covidence.org) to determine whether an article would be subsequently reviewed in full-text format. The following criteria were applied: The study presented original empirical results, was published in a peer-reviewed journal, was written in English or German, included adult healthy participants, and an explicit emotion regulation paradigm was assessed where participants are explicitly told to use emotion regulation strategies to modulate an emotion. We discarded studies that did not assess a psychophysiological measure of interest (e.g., EEG studies) at this point. Based on these criteria, the same two reviewers independently reviewed 157 studies in full-text format.

### Inclusion/Exclusion Criteria

The 157 studies were examined to determine if they met the following inclusion criteria of our analysis: The study (1) included a control condition in which participants were confronted with emotional contents but did not regulate emotions (see Table 1 for definitions of possible control instructions), (2) sampled a psychophysiological measure throughout the regulation phases, (3) did not assess an experimental intervention before the emotion regulation task that may influence the performance of emotion regulation, (4) provided sufficient information to compute the effect size, (5) induced negative emotions, (6) instructed participants to use one or more of the strategies provided in Table 1. If studies met inclusion criteria (1) to (6) but did not provide adequate information for effect size computation, we asked the authors for the needed information via e-mail.

**Figure 1:**
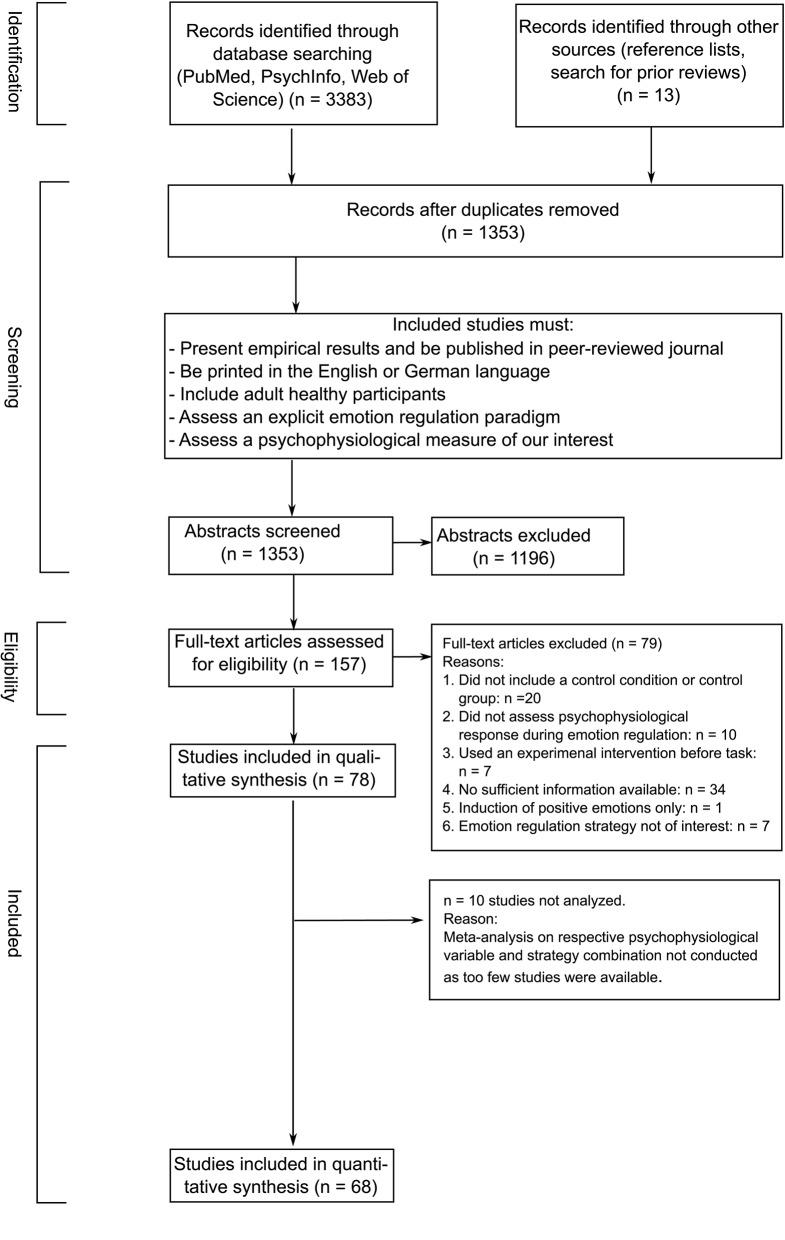
PRISMA flowchart of the literature search process.

Finally, a total of *n* = 78 studies fulfilled all inclusion criteria. Of those, *n* = 68 entered our quantitative synthesis (for an overview see Table 3). The remaining 10 studies (Delgado et al., 2008; Driscoll et al., 2009; Jamieson et al., 2012, 2013; Peters et al., 2014; Baur et al., 2015; Reinecke et al., 2015; Peters and Jamieson, 2016; Zaehringer et al., 2018; Kotwas et al., 2019) were not considered, as a meta-analysis on the respective combination of emotion regulation strategy and psychophysiological measure was not possible because the number of studies was too small. See Figure 1 for a PRISMA flowchart depiction of the screening and selection of studies.

**Table 3:** Characteristics and effect sizes for studies included in the meta–analyses.

**Study name**	**Strategy**	**Measure**	**Emotion**	**Design**	**Trial duration (s)**	**Nature of emotion induction**	**Control instruction**	**N total**	**Percent of women**	**Age (mean)**	**N analyzed**	**Effect size**
Ajaya et al. (2016)	Reappraisal	HRV	Anger	B	120	Anger task	C1	66	60.61	20.62	40	−0.10
Aldao and Mennin (2012)	Reappraisal	HRV	Disgust, fear, sadness	B	62	F	C1	58	56.90	29.57	38	0.75
Azbel-Jackson et al. (2015), study 1	Suppression	HR	Negative	B	7	I	C2	60	70.00	21.50	60	−0.22
Azbel-Jackson et al. (2015), study 1	Suppression	SCL	Negative	B	7	I	C2	60	70.00	21.50	60	−0.04
Azbel-Jackson et al. (2015), study 2	Suppression	HR	Negative	B	7	I	C2	80	85.00	22.20	40	0.40
Azbel-Jackson et al. (2015), study 2	Suppression	SCL	Negative	B	7	I	C2	80	85.00	22.20	40	0.73
Bebko et al. (2011)	Reappraisal	PD	Negative	W	10	I	C4	84	47.62	19.67	40	−0.09
Ben-Naim et al. (2013)	Reappraisal	FPA	Negative	B	900	Dyadic	C1	254	50.00	24.00	86	−1.52
Ben-Naim et al. (2013)	Reappraisal	FPTT	Negative	B	900	Dyadic	C1	254	50.00	24.00	86	−0.18
Ben-Naim et al. (2013)	Reappraisal	HR	Negative	B	900	Dyadic	C1	254	50.00	24.00	86	0.33
Ben-Naim et al. (2013)	Reappraisal	SCL	Negative	B	900	Dyadic	C1	254	50.00	24.00	86	0.16
Ben-Naim et al. (2013)	Reappraisal	SCR	Negative	B	900	Dyadic	C1	254	50.00	24.00	86	−0.39
Ben-Naim et al. (2013)	Suppression	EPPT	Negative	B	900	Dyadic	C1	254	50.00	24.00	85	0.09
Ben-Naim et al. (2013)	Suppression	FPA	Negative	B	900	Dyadic	C1	254	50.00	24.00	85	−0.66
Ben-Naim et al. (2013)	Suppression	FPTT	Negative	B	900	Dyadic	C1	254	50.00	24.00	85	−0.32
Ben-Naim et al. (2013)	Suppression	HR	Negative	B	900	Dyadic	C1	254	50.00	24.00	85	0.35
Ben-Naim et al. (2013)	Suppression	SCL	Negative	B	900	Dyadic	C1	254	50.00	24.00	85	0.04
Braams et al. (2012)	Suppression	HR	Fear	B	16.5	ToS	C1	123	46.34	21.70	62	−0.04
Bulut et al. (2018), study 1	Reappraisal	HRV	Negative	B	300	I	C4	28	67.86	23.67	28	0.47
Butler et al. (2003), study 1	Suppression	MAP	Negative	B		Dyadic	C1	72	100.00	20.30	60	−0.09
Butler et al. (2006)	Reappraisal	HR	Negative	B	590.8	Dyadic	C1	190	100.00	20.00	62	−0.24
Butler et al. (2006)	Reappraisal	HRV	Negative	B	590.8	Dyadic	C1	190	100.00	20.00	62	0.51
Butler et al. (2006)	Reappraisal	RA	Negative	B	590.8	Dyadic	C1	190	100.00	20.00	62	0.12
Butler et al. (2006)	Suppression	HR	Negative	B	570.6	Dyadic	C1	190	100.00	20.00	69	0.10
Butler et al. (2006)	Suppression	HRV	Negative	B	570.6	Dyadic	C1	190	100.00	20.00	69	0.39
Butler et al. (2006)	Suppression	RA	Negative	B	570.6	Dyadic	C1	190	100.00	20.00	69	−0.76
Butler et al. (2014)	Reappraisal	SCL	Negative	B	590.8	Dyadic	C1	190	14.74	20.10	61	−0.28
Butler et al. (2014)	Suppression	SCL	Negative	B	570.6	Dyadic	C1	190	14.74	20.10	68	−0.26
Chu et al. (2019)	Reappraisal	HR	Anger	B	10	Anger task	C1	68	54.41	40.00	68	−0.14
Colby et al. (1977)	Suppression	SCL	Fear	W	6	ToS	C4	10	0.00		10	−0.11
Conzelmann et al. (2015)	Own choice	Startle	Negative	W	8	I	C3	31	48.39	22.00	31	−0.60
Dan-Glauser and Gross (2011)	Suppression	FT	Negative	W	8	I	C4	37	100.00	20.20	37	−0.16
Dan-Glauser and Gross (2011)	Suppression	HR	Negative	W	8	I	C4	37	100.00	20.20	37	−0.57
Dan-Glauser and Gross (2011)	Suppression	MAP	Negative	W	8	I	C4	37	100.00	20.20	37	−0.07
Dan-Glauser and Gross (2011)	Suppression	RA	Negative	W	8	I	C4	37	100.00	20.20	37	−0.82
Dan-Glauser and Gross (2015)	Suppression	FPA	Negative	W	8	I	C4	37	100.00	20.20	37	0.42
Dan-Glauser and Gross (2015)	Suppression	FPTT	Negative	W	8	I	C4	37	100.00	20.20	37	−0.13
Dan-Glauser and Gross (2015)	Suppression	FT	Negative	W	8	I	C4	37	100.00	20.20	37	−0.16
Dan-Glauser and Gross (2015)	Suppression	HR	Negative	W	8	I	C4	37	100.00	20.20	37	−0.71
Dan-Glauser and Gross (2015)	Suppression	MAP	Negative	W	8	I	C4	37	100.00	20.20	37	−0.50
Dan-Glauser and Gross (2015)	Suppression	RA	Negative	W	8	I	C4	37	100.00	20.20	37	−0.45
Demaree et al. (2006)	Suppression	HR	Disgust	B	120	F	C4	69	52.17	19.32	35	0.09
Demaree et al. (2006)	Suppression	HRV	Disgust	B	120	F	C4	69	52.17	19.32	35	0.21
Demaree et al. (2006)	Suppression	RA	Disgust	B	120	F	C4	69	52.17	19.32	35	0.43
Demaree et al. (2006)	Suppression	SCL	Disgust	B	120	F	C4	69	52.17	19.32	35	0.12
Denson et al. (2014), study 1	Reappraisal	HR	Fear	B	600	Stress	C1	90	52.22	20.54	90	−0.09
Denson et al. (2014), study 1	Reappraisal	HR	Fear	B	300	Stress	C1	90	52.22	20.54	86	−0.07
Denson et al. (2011)	Reappraisal	HRV	Anger	B	180	F	C1	131	100.00	20.23	86	0.37
Denson et al. (2011)	Suppression	HR	Anger	B	180	F	C1	131	100.00	20.23	89	0.25
Denson et al. (2011)	Suppression	HRV	Anger	B	180	F	C1	131	100.00	20.23	89	0.17
Deveney and Pizzagalli (2008)	Reappraisal	cEMG	Negative	W	5	I	C3	32	78.13	23.97	26	−0.09
Di Simplicio et al. (2012), sample 1	Reappraisal	HR	Negative	W	4	I	C4	30	53.33	28.59	20	0.00
Di Simplicio et al. (2012), sample 1	Reappraisal	HRV	Negative	W	4	I	C4	30	53.33	28.59	20	0.05
Di Simplicio et al. (2012), sample 2	Reappraisal	HR	Negative	W	4	I	C4	30	53.33	28.59	10	0.09
Di Simplicio et al. (2012), sample 2	Reappraisal	HRV	Negative	W	4	I	C4	30	53.33	28.59	10	−0.15
Dillon and LaBar (2005), sample 1	Own choice	Startle	Negative	W	12	I	C3	48	77.08	22.00	12	−0.09
Dillon and LaBar (2005), sample 2	Own choice	Startle	Negative	W	12	I	C3	48	77.08	22.00	12	−0.75
Efinger et al. (2019)	Reappraisal	HR	Negative	W	8	I	C4	77	100.00	20.70	77	−0.27
Efinger et al. (2019)	Reappraisal	RA	Negative	W	8	I	C4	77	100.00	20.70	77	0.06
Efinger et al. (2019)	Reappraisal	SCL	Negative	W	8	I	C4	77	100.00	20.70	77	−0.19
Efinger et al. (2019)	Distraction	SCL	Negative	W	8	I	C4	77	100.00	20.70	77	−0.27
Fitzpatrick and Kuo (2016)	Distraction	SCL	Negative	W	10	I		30	66.67	30.07	30	0.00
Fuentes-Sánchez et al. (2019)	Reappraisal	SCR	Negative	W	8	I	C4	122	59.02	25.10	106	−0.01
Goldin et al. (2019)	Reappraisal	HR	Negative	W	12	Self-belief	C4	35	57.14	32.20	35	−0.03
Goldin et al. (2019)	Reappraisal	SCL	Negative	W	12	Self-belief	C4	35	57.14	32.20	35	−0.01
Golkar et al. (2014)	Own choice	Startle	Negative	W	5	I	C2	61	54.10	30.90	61	−0.47
Gomez et al. (2015)	Reappraisal	SCR	Disgust	B	10	I	C1	81	64.20	28.15	40	−0.11
Gross and Levenson (1993), study 1	Suppression	EPPT	Disgust	B	64	F	C1	43	0.00	19.30	43	0.07
Gross and Levenson (1993), study 1	Suppression	FPA	Disgust	B	64	F	C1	43	0.00	19.30	43	−0.38
Gross and Levenson (1993), study 1	Suppression	FPTT	Disgust	B	64	F	C1	43	0.00	19.30	43	−0.24
Gross and Levenson (1993), study 1	Suppression	FT	Disgust	B	64	F	C1	43	0.00	19.30	43	−0.30
Gross and Levenson (1993), study 1	Suppression	HR	Disgust	B	64	F	C1	43	0.00	19.30	43	−0.53
Gross and Levenson (1993), study 1	Suppression	RA	Disgust	B	64	F	C1	43	0.00	19.30	43	−0.18
Gross and Levenson (1993), study 1	Suppression	SCL	Disgust	B	64	F	C1	43	0.00	19.30	43	0.24
Gross and Levenson (1993), study 2	Suppression	EPPT	Disgust	B	64	F	C1	42	100.00	19.20	42	−0.55
Gross and Levenson (1993), study 2	Suppression	FPA	Disgust	B	64	F	C1	42	100.00	19.20	42	−0.81
Gross and Levenson (1993), study 2	Suppression	FPTT	Disgust	B	64	F	C1	42	100.00	19.20	42	0.21
Gross and Levenson (1993), study 2	Suppression	FT	Disgust	B	64	F	C1	42	100.00	19.20	42	−0.96
Gross and Levenson (1993), study 2	Suppression	HR	Disgust	B	64	F	C1	42	100.00	19.20	42	−0.21
Gross and Levenson (1993), study 2	Suppression	RA	Disgust	B	64	F	C1	42	100.00	19.20	42	0.11
Gross and Levenson (1993), study 2	Suppression	SCL	Disgust	B	64	F	C1	42	100.00	19.20	42	0.46
Gross and Levenson (1997)	Suppression	SCL	Sadness	B	210	F	C1	180	100.00		180	0.29
Gross (1998a)	Reappraisal	FPA	Disgust	B	64	F	C1	120	50.00	21.00	80	0.12
Gross (1998a)	Reappraisal	FT	Disgust	B	64	F	C1	120	50.00	21.00	80	−0.33
Gross (1998a)	Reappraisal	HR	Disgust	B	64	F	C1	120	50.00	21.00	80	−0.09
Gross (1998a)	Reappraisal	SCL	Disgust	B	64	F	C1	120	50.00	21.00	80	−0.19
Gross (1998a)	Suppression	FPA	Disgust	B	64	F	C1	120	50.00	21.00	80	−0.60
Gross (1998a)	Suppression	FT	Disgust	B	64	F	C1	120	50.00	21.00	80	−1.04
Gross (1998a)	Suppression	HR	Disgust	B	64	F	C1	120	50.00	21.00	80	0.02
Gross (1998a)	Suppression	SCL	Disgust	B	64	F	C1	120	50.00	21.00	80	0.41
Hagemann et al. (2006)	Suppression	EPPT	Negative	B	5	ToS, I	C1	252	51.98	20.50	168	−0.38
Hagemann et al. (2006)	Suppression	FPA	Negative	B	5	ToS, I	C1	252	51.98	20.50	168	−0.25
Hagemann et al. (2006)	Suppression	FPTT	Negative	B	5	ToS, I	C1	252	51.98	20.50	168	−0.39
Hagemann et al. (2006)	Suppression	FT	Negative	B	5	ToS, I	C1	252	51.98	20.50	168	−0.55
Hagemann et al. (2006)	Suppression	HR	Negative	B	5	ToS, I	C1	252	51.98	20.50	168	0.73
Hagemann et al. (2006)	Suppression	HRV	Negative	B	20	ToS, I	C1	252	51.98	20.50	168	−0.34
Hagemann et al. (2006)	Suppression	SCL	Negative	B	5	ToS, I	C1	252	51.98	20.50	168	0.49
Hallam et al. (2015)	Reappraisal	SCL	Negative	W	10	I	C4	40	50.00	20.00	26	0.00
Hallam et al. (2015)	Suppression	SCL	Negative	W	10	I	C4	40	50.00	20.00	26	−0.01
Jackson et al. (2000)	Own choice	Startle	Negative	W	14	I	C3	48	68.75	20.50	44	−1.04
Kim and Hamann (2012)	Reappraisal	cEMG	Negative	W	24	I	C4	36	50.00	20.19	33	−0.30
Kim and Hamann (2012)	Reappraisal	SCR	Negative	W	24	I	C4	36	50.00	20.19	32	0.11
Kinner et al. (2017)	Reappraisal	PD	Negative	W	5	I	C4	30	100.00	24.40	28	0.26
Kinner et al. (2017)	Reappraisal	SCR	Negative	W	5	I	C4	30	100.00	24.40	25	0.00
Kunzmann et al. (2005)	Suppression	HR	Disgust	W	117	F	C1	95	49.47	46.00	47	−0.26
Kunzmann et al. (2005)	Suppression	SCL	Disgust	W	117	F	C1	95	49.47	46.00	47	0.15
Leiberg et al. (2012)	Reappraisal	SCR	Negative	W	6	I	C4	24	100.00	24.10	24	0.17
Lohani and Isaacowitz (2014), sample 1	Reappraisal	cEMG	Sadness	W	300	F	C1	48	79.17	71.42	42	−0.17
Lohani and Isaacowitz (2014), sample 1	Reappraisal	SCL	Sadness	W	300	F	C1	42	73.81	18.50	40	0.56
Lohani and Isaacowitz (2014), sample 1	Suppression	SCL	Sadness	W	300	F	C1	42	73.81	18.50	40	0.52
Lohani and Isaacowitz (2014), sample 2	Reappraisal	cEMG	Sadness	W	300	F	C1	42	73.81	18.50	40	−0.30
Lohani and Isaacowitz (2014), sample 2	Reappraisal	SCL	Sadness	W	300	F	C1	48	79.17	71.42	44	0.09
Lohani and Isaacowitz (2014), sample 2	Suppression	SCL	Sadness	W	300	F	C1	48	79.17	71.42	44	0.13
Lohani and Isaacowitz (2014), sample 1	Distraction	SCL	Sadness	W	300	F	C1	42	73.81	18.50	40	0.48
Lohani and Isaacowitz (2014), sample 2	Distraction	SCL	Sadness	W	300	F	C1	48	79.17	71.42	44	0.24
Low et al. (2008)	Reappraisal	HR	Negative	B	600	Stress	C3	81	58.02	20.60	56	0.29
Martins et al. (2018)	Reappraisal	PD	Negative	W	7	I	C4	48	68.75	69.10	48	0.06
Martins et al. (2018)	Reappraisal	PD	Negative	W	7	I	C4	48	60.42	21.06	48	0.06
Morawetz et al. (2016a)	Reappraisal	SCR	Negative	W	8	I, F	C4	59	33.90	32.47	47	0.08
Morawetz et al. (2016b)	Reappraisal	SCR	Negative	W	8	I	C4	23	52.17	25.70	16	−0.19
Morawetz et al. (2017)	Reappraisal	SCR	Negative	W	8	F	C4	23	65.22	22.95	22	−0.03
Ohira et al. (2006)	Suppression	HR	Negative	W	60	I	C4	10	100.00	24.22	9	0.04
Opitz et al. (2014), sample 1	Reappraisal	cEMG	Sadness	W	8	I	C4	30	53.33	61.90	29	−0.43
Opitz et al. (2014), sample 1	Reappraisal	HR	Sadness	W	8	I	C4	30	63.33	19.45	28	−0.02
Opitz et al. (2014), sample 1	Reappraisal	SCL	Sadness	W	8	I	C4	30	63.33	19.45	27	−0.02
Opitz et al. (2014), sample 2	Reappraisal	cEMG	Sadness	W	8	I	C4	30	63.33	19.45	28	−1.07
Opitz et al. (2014), sample 2	Reappraisal	HR	Sadness	W	8	I	C4	30	53.33	61.90	29	−0.14
Opitz et al. (2014), sample 2	Reappraisal	SCL	Sadness	W	8	I	C4	30	53.33	61.90	29	−0.27
Ortner (2015)	Reappraisal	SCR	Negative	B	8	I	C1	120	75.83		76	0.01
Plieger et al. (2017)	Reappraisal	SCL	Negative	W	4.5	I	C1	91	82.42	24.53	91	−0.28
Richards and Gross (1999), study2	Suppression	DBP	Negative	B	84	I	C1	85	100.00	18.80	74	0.36
Richards and Gross (1999), study2	Suppression	FT	Negative	B	84	I	C1	85	100.00	18.80	74	−0.37
Richards and Gross (1999), study2	Suppression	HR	Negative	B	84	I	C1	85	100.00	18.80	74	−0.11
Richards and Gross (1999), study2	Suppression	SBP	Negative	B	84	I	C1	85	100.00	18.80	74	0.27
Richards and Gross (1999), study2	Suppression	SCL	Negative	B	84	I	C1	85	100.00	18.80	74	−0.14
Roberts et al. (2008), sample 1	Suppression	DBP	Disgust	B	62	F	C1	40	60.00	20.80	40	0.91
Roberts et al. (2008), sample 1	Suppression	HR	Disgust	B	62	F	C1	40	60.00	20.80	40	−0.23
Roberts et al. (2008), sample 1	Suppression	SBP	Disgust	B	62	F	C1	40	60.00	20.80	40	0.60
Roberts et al. (2008), sample 1	Suppression	SCL	Negative	B	62	F	C1	40	60.00	20.80	40	0.00
Roberts et al. (2008), sample 2	Suppression	DBP	Disgust	B	62	F	C1	40	60.00	20.80	40	0.84
Roberts et al. (2008), sample 2	Suppression	HR	Disgust	B	62	F	C1	40	60.00	20.80	40	0.08
Roberts et al. (2008), sample 2	Suppression	SBP	Disgust	B	62	F	C1	40	60.00	20.80	40	0.66
Roberts et al. (2008), sample 2	Suppression	SCL	Negative	B	62	F	C1	40	60.00	20.80	40	0.35
Roberts et al. (2008), sample 3	Suppression	DBP	Disgust	B	62	F	C1	40	60.00	20.80	40	−0.31
Roberts et al. (2008), sample 3	Suppression	HR	Disgust	B	62	F	C1	40	60.00	20.80	40	−0.61
Roberts et al. (2008), sample 3	Suppression	SBP	Disgust	B	62	F	C1	40	60.00	20.80	40	0.01
Roberts et al. (2008), sample 3	Suppression	SCL	Negative	B	62	F	C1	40	60.00	20.80	40	0.62
Roberts et al. (2008), sample 4	Suppression	DBP	Disgust	B	62	F	C1	40	60.00	20.80	40	0.12
Roberts et al. (2008), sample 4	Suppression	HR	Disgust	B	62	F	C1	40	60.00	20.80	40	0.26
Roberts et al. (2008), sample 4	Suppression	SBP	Disgust	B	62	F	C1	40	60.00	20.80	40	0.11
Roberts et al. (2008), sample 4	Suppression	SCL	Negative	B	62	F	C1	40	60.00	20.80	40	0.30
Robinson and Demaree (2009)	Suppression	HR	Sadness	W	120	F	C4	102	50.98	19.75	102	−0.23
Robinson and Demaree (2009)	Suppression	HRV	Sadness	W	120	F	C4	102	50.98	19.75	102	0.41
Robinson and Demaree (2009)	Suppression	SCL	Sadness	W	120	F	C4	102	50.98	19.75	102	0.26
Rohrmann et al. (2009), sample 1	Reappraisal	HR	Disgust	B	60	F	C1	120	0.00	25.47	36	0.22
Rohrmann et al. (2009), sample 2	Reappraisal	HR	Disgust	B	60	F	C1	120	0.00	25.47	36	−0.34
Rohrmann et al. (2009), sample 1	Suppression	HR	Disgust	B	60	F	C1	120	0.00	25.47	36	0.47
Rohrmann et al. (2009), sample 2	Suppression	HR	Disgust	B	60	F	C1	120	0.00	25.47	36	−0.66
Rohrmann et al. (2009), sample 1	Reappraisal	SCL	Disgust	B	60	F	C1	120	0.00	25.47	36	0.35
Rohrmann et al. (2009), sample 2	Reappraisal	SCL	Disgust	B	60	F	C1	120	0.00	25.47	36	−0.57
Rohrmann et al. (2009), sample 1	Suppression	SCL	Disgust	B	60	F	C1	120	0.00	25.47	36	0.85
Rohrmann et al. (2009), sample 2	Suppression	SCL	Disgust	B	60	F	C1	120	0.00	25.47	36	−0.23
Roth et al. (2014), study2	Suppression	SCL	Fear	B	197	F	C1	116	60.34	24.90	65	−0.04
Roth et al. (2014), study2	Distraction	SCL	Fear	B	197	F	C1	116	60.34	24.90	67	−0.77
Sheppes et al. (2009)	Reappraisal	FT	Sadness	B	190	F	C5	45	100.00	22.90	29	0.22
Sheppes et al. (2009)	Reappraisal	SCL	Sadness	B	190	F	C5	45	100.00	22.90	29	1.13
Sheppes et al. (2009)	Distraction	SCL	Sadness	B	190	F	C5	45	100.00	22.90	29	0.23
Shermohammed et al. (2017)	Reappraisal	HR	Negative	W	8	I	C1	25	48.00	20.89	19	0.65
Shermohammed et al. (2017)	Reappraisal	SCR	Negative	W	8	I	C1	25	48.00	20.89	17	0.12
Shiota and Levenson (2009, 2012), sample 1	Suppression	DBP	Disgust	W	180	F	C4	76	50.00	25.50	73	−0.66
Shiota and Levenson (2009, 2012), sample 1	Suppression	EPPT	Disgust	W	180	F	C4	76	50.00	25.50	74	0.33
Shiota and Levenson (2009, 2012), sample 1	Suppression	FPA	Disgust	W	180	F	C4	76	50.00	25.50	75	0.49
Shiota and Levenson (2009, 2012), sample 1	Suppression	FPTT	Disgust	W	180	F	C4	76	50.00	25.50	75	−0.12
Shiota and Levenson (2009, 2012), sample 1	Suppression	FT	Disgust	W	180	F	C4	76	50.00	25.50	76	−0.24
Shiota and Levenson (2009, 2012), sample 1	Suppression	HR	Disgust	W	180	F	C4	76	50.00	25.50	75	−0.40
Shiota and Levenson (2009, 2012), sample 1	Suppression	MAP	Disgust	W	180	F	C4	76	50.00	25.50	73	−0.66
Shiota and Levenson (2009, 2012), sample 1	Suppression	RA	Disgust	W	180	F	C4	76	50.00	25.50	72	−0.29
Shiota and Levenson (2009, 2012), sample 1	Suppression	SBP	Disgust	W	180	F	C4	76	50.00	25.50	73	−0.69
Shiota and Levenson (2009, 2012), sample 1	Suppression	SCL	Disgust	W	180	F	C4	76	50.00	25.50	73	−0.42
Shiota and Levenson (2009, 2012), sample 2	Reappraisal	FPA	Disgust, sadness	W	180	F	C4	22	50.00	25.50	23	0.37
Shiota and Levenson (2009, 2012), sample 2	Reappraisal	FPTT	Disgust, sadness	W	180	F	C4	22	50.00	25.50	23	0.47
Shiota and Levenson (2009, 2012), sample 2	Reappraisal	FT	Disgust, sadness	W	180	F	C4	22	50.00	25.50	23	0.36
Shiota and Levenson (2009, 2012), sample 2	Reappraisal	HR	Disgust, sadness	W	180	F	C4	22	50.00	25.50	23	−0.29
Shiota and Levenson (2009, 2012), sample 2	Reappraisal	RA	Disgust, sadness	W	180	F	C4	22	50.00	25.50	22	−0.34
Shiota and Levenson (2009, 2012), sample 2	Reappraisal	SCL	Disgust, sadness	W	180	F	C4	22	50.00	25.50	23	−0.27
Shiota and Levenson (2009, 2012), sample 3	Reappraisal	FPA	Disgust, sadness	W	180	F	C4	26	50.00	25.30	25	0.14
Shiota and Levenson (2009, 2012), sample 3	Reappraisal	FPTT	Disgust, sadness	W	180	F	C4	26	50.00	25.30	25	0.02
Shiota and Levenson (2009, 2012), sample 3	Reappraisal	FT	Disgust, sadness	W	180	F	C4	26	50.00	25.30	26	0.12
Shiota and Levenson (2009, 2012), sample 3	Reappraisal	HR	Disgust, sadness	W	180	F	C4	26	50.00	25.30	25	−0.11
Shiota and Levenson (2009, 2012), sample 3	Reappraisal	RA	Disgust, sadness	W	180	F	C4	26	50.00	25.30	24	−0.10
Shiota and Levenson (2009, 2012), sample 3	Reappraisal	SCL	Disgust, sadness	W	180	F	C4	26	50.00	25.30	24	0.10
Shiota and Levenson (2009, 2012), sample 4	Suppression	DBP	Disgust	W	180	F	C4	72	50.00	44.70	64	−0.27
Shiota and Levenson (2009, 2012), sample 4	Suppression	EPPT	Disgust	W	180	F	C4	72	50.00	44.70	71	−0.06
Shiota and Levenson (2009, 2012), sample 4	Suppression	FPA	Disgust	W	180	F	C4	72	50.00	44.70	71	0.27
Shiota and Levenson (2009, 2012), sample 4	Suppression	FPTT	Disgust	W	180	F	C4	72	50.00	44.70	72	0.11
Shiota and Levenson (2009, 2012), sample 4	Suppression	FT	Disgust	W	180	F	C4	72	50.00	44.70	72	−0.03
Shiota and Levenson (2009, 2012), sample 4	Suppression	HR	Disgust	W	180	F	C4	72	50.00	44.70	72	−0.30
Shiota and Levenson (2009, 2012), sample 4	Suppression	MAP	Disgust	W	180	F	C4	72	50.00	44.70	64	−0.28
Shiota and Levenson (2009, 2012), sample 4	Suppression	RA	Disgust	W	180	F	C4	72	50.00	44.70	66	−0.07
Shiota and Levenson (2009, 2012), sample 4	Suppression	SBP	Disgust	W	180	F	C4	72	50.00	44.70	64	−0.32
Shiota and Levenson (2009, 2012), sample 4	Suppression	SCL	Disgust	W	180	F	C4	72	50.00	44.70	69	−0.39
Shiota and Levenson (2009, 2012), sample 5	Reappraisal	FPA	Disgust, sadness	W	180	F	C4	22	50.00	44.70	23	0.23
Shiota and Levenson (2009, 2012), sample 5	Reappraisal	FPTT	Disgust, sadness	W	180	F	C4	22	50.00	44.70	24	−0.28
Shiota and Levenson (2009, 2012), sample 5	Reappraisal	FT	Disgust, sadness	W	180	F	C4	22	50.00	44.70	24	0.00
Shiota and Levenson (2009, 2012), sample 5	Reappraisal	HR	Disgust, sadness	W	180	F	C4	22	50.00	44.70	24	−0.31
Shiota and Levenson (2009, 2012), sample 5	Reappraisal	RA	Disgust, sadness	W	180	F	C4	22	50.00	44.70	23	−0.18
Shiota and Levenson (2009, 2012), sample 5	Reappraisal	SCL	Disgust, sadness	W	180	F	C4	22	50.00	44.70	22	−0.10
Shiota and Levenson (2009, 2012), sample 6	Reappraisal	FPA	Disgust, sadness	W	180	F	C4	26	50.00	43.20	26	0.17
Shiota and Levenson (2009, 2012), sample 6	Reappraisal	FPTT	Disgust, sadness	W	180	F	C4	26	50.00	43.20	26	0.21
Shiota and Levenson (2009, 2012), sample 6	Reappraisal	FT	Disgust, sadness	W	180	F	C4	26	50.00	43.20	26	0.23
Shiota and Levenson (2009, 2012), sample 6	Reappraisal	HR	Disgust, sadness	W	180	F	C4	26	50.00	43.20	26	−0.06
Shiota and Levenson (2009, 2012), sample 6	Reappraisal	RA	Disgust, sadness	W	180	F	C4	26	50.00	43.20	24	−0.10
Shiota and Levenson (2009, 2012), sample 6	Reappraisal	SCL	Disgust, sadness	W	180	F	C4	26	50.00	43.20	25	−0.09
Shiota and Levenson (2009, 2012), sample 7	Suppression	DBP	Disgust	W	180	F	C4	72	50.00	64.80	69	−0.30
Shiota and Levenson (2009, 2012), sample 7	Suppression	EPPT	Disgust	W	180	F	C4	72	50.00	64.80	68	−0.01
Shiota and Levenson (2009, 2012), sample 7	Suppression	FPA	Disgust	W	180	F	C4	72	50.00	64.80	65	0.23
Shiota and Levenson (2009, 2012), sample 7	Suppression	FPTT	Disgust	W	180	F	C4	72	50.00	64.80	65	0.16
Shiota and Levenson (2009, 2012), sample 7	Suppression	FT	Disgust	W	180	F	C4	72	50.00	64.80	72	0.11
Shiota and Levenson (2009, 2012), sample 7	Suppression	HR	Disgust	W	180	F	C4	72	50.00	64.80	69	−0.12
Shiota and Levenson (2009, 2012), sample 7	Suppression	MAP	Disgust	W	180	F	C4	72	50.00	64.80	69	−0.30
Shiota and Levenson (2009, 2012), sample 7	Suppression	RA	Disgust	W	180	F	C4	72	50.00	64.80	66	−0.26
Shiota and Levenson (2009, 2012), sample 7	Suppression	SBP	Disgust	W	180	F	C4	72	50.00	64.80	69	−0.27
Shiota and Levenson (2009, 2012), sample 7	Suppression	SCL	Disgust	W	180	F	C4	72	50.00	64.80	69	−0.46
Shiota and Levenson (2009, 2012), sample 8	Reappraisal	FPA	Disgust, sadness	W	180	F	C4	24	50.00	64.80	23	−0.08
Shiota and Levenson (2009, 2012), sample 8	Reappraisal	FPTT	Disgust, sadness	W	180	F	C4	24	50.00	64.80	23	0.03
Shiota and Levenson (2009, 2012), sample 8	Reappraisal	FT	Disgust, sadness	W	180	F	C4	24	50.00	64.80	24	0.10
Shiota and Levenson (2009, 2012), sample 8	Reappraisal	HR	Disgust, sadness	W	180	F	C4	24	50.00	64.80	23	−0.19
Shiota and Levenson (2009, 2012), sample 8	Reappraisal	RA	Disgust, sadness	W	180	F	C4	24	50.00	64.80	20	−0.19
Shiota and Levenson (2009, 2012), sample 8	Reappraisal	SCL	Disgust, sadness	W	180	F	C4	24	50.00	64.80	23	−0.11
Shiota and Levenson (2009, 2012), sample 9	Reappraisal	FPA	Disgust, sadness	W	180	F	C4	24	50.00	64.50	22	0.40
Shiota and Levenson (2009, 2012), sample 9	Reappraisal	FPTT	Disgust, sadness	W	180	F	C4	24	50.00	64.50	22	−0.12
Shiota and Levenson (2009, 2012), sample 9	Reappraisal	FT	Disgust, sadness	W	180	F	C4	24	50.00	64.50	23	0.58
Shiota and Levenson (2009, 2012), sample 9	Reappraisal	HR	Disgust, sadness	W	180	F	C4	24	50.00	64.50	22	−0.10
Shiota and Levenson (2009, 2012), sample 9	Reappraisal	RA	Disgust, sadness	W	180	F	C4	24	50.00	64.50	22	−0.26
Shiota and Levenson (2009, 2012), sample 9	Reappraisal	SCL	Disgust, sadness	W	180	F	C4	24	50.00	64.50	22	−0.64
Soto et al. (2016)	Suppression	HR	Disgust	W	58	F	C1	59	54.24	19.51	48	−0.19
Soto et al. (2016)	Suppression	SCL	Disgust	W	58	F	C1	59	54.24	19.51	47	−0.15
Stiller et al. (2019)	Reappraisal	HR	Negative	B	165	F	C2	61	73.77	24.30	41	0.15
Stiller et al. (2019)	Reappraisal	SCL	Negative	B	165	F	C2	61	73.77	24.30	41	0.49
Stiller et al. (2019)	Suppression	HR	Negative	B	165	F	C2	61	73.77	24.30	40	0.58
Stiller et al. (2019)	Suppression	SCL	Negative	B	165	F	C2	61	73.77	24.30	40	0.35
Strauss et al. (2016)	Reappraisal	PD	Negative	W	5	I	C4	25	64.00	19.80	25	0.14
Svaldi et al. (2010)	Reappraisal	FPTT	Sadness	W	125	F	C1	25	100.00	38.30	21	−0.11
Svaldi et al. (2010)	reappraisal	HR	Sadness	W	125	F	C1	25	100.00	38.30	25	−0.32
Svaldi et al. (2010)	reappraisal	HRV	Sadness	W	125	F	C1	25	100.00	38.30	21	−0.67
Svaldi et al. (2010)	reappraisal	SCL	Sadness	W	125	F	C1	25	100.00	38.30	23	0.10
Svaldi et al. (2010)	Suppression	FPTT	Sadness	W	211	F	C1	25	100.00	38.30	21	−0.68
Svaldi et al. (2010)	Suppression	HR	Sadness	W	211	F	C1	25	100.00	38.30	25	−0.16
Svaldi et al. (2010)	Suppression	HRV	Sadness	W	211	F	C1	25	100.00	38.30	21	−0.18
Svaldi et al. (2010)	Suppression	SCL	Sadness	W	211	F	C1	25	100.00	38.30	23	0.50
Urry et al. (2006)	Reappraisal	PD	Negative	W	5	I	C3	17	52.94	62.90	14	0.43
Urry et al. (2009)	Reappraisal	PD	Negative	W	8	I	C3	26	57.69	64.80	26	0.46
Urry et al. (2009)	Reappraisal	SCL	Negative	W	8	I	C3	26	57.69	64.80	26	−0.42
Urry (2009)	Reappraisal	cEMG	Negative	W	8	I	C2	41	63.41	20.00	40	0.03
Urry (2009)	Reappraisal	HR	Negative	W	8	I	C2	41	63.41	20.00	40	−0.14
Urry (2009)	Reappraisal	SCR	Negative	W	8	I	C2	41	63.41	20.00	39	0.18
Urry (2010)	Reappraisal	cEMG	Negative	W	4	I	C4	54	48.15	18.80	54	−0.32
Urry (2010)	Reappraisal	HR	Negative	W	4	I	C4	54	48.15	18.80	53	0.03
Urry (2010)	Reappraisal	SCL	Negative	W	4	I	C4	54	48.15	18.80	52	0.03
Uy et al. (2013)	Suppression	HRV	Disgust	W	133	F	C1	7	57.14	29.80	7	0.45
van Reekum et al. (2007)	Reappraisal	PD	Negative	W	8	I	C4	29	62.07	63.00	21	0.53
Williams et al. (2009), study 1	Reappraisal	HR	Fear	B	180	Stress	C1	39	64.10	20.60	26	−0.15
Williams et al. (2009), study 2	Reappraisal	HR	Fear	B	180	Stress	C1	47	65.96	21.30	30	0.05
Wolgast et al. (2011)	Reappraisal	cEMG	Disgust, fear, Sadness	B	153	F	C1	94	51.06	27.40	62	−0.79
Wolgast et al. (2011)	Reappraisal	SCL	Disgust, fear, Sadness	B	153	F	C1	94	51.06	27.40	62	−0.87
Wu et al. (2016), study 2	Reappraisal	SCL	Sadness	B	180	F	C1	42	100.00	22.31	42	−0.18
Yuan et al. (2014)	Suppression	SCL	Anger	B	1800	Anger task	C1	64	0.00	29.52	43	−0.72

*cEMG, corrugator electromyography; DBP, diastolic blood pressure; EPTT, ear pulse transit time; FPA, finger pulse amplitude; FPTT, finger pulse transit time; FT, finger temperature; HR, heart rate; HRV, heart rate variability; MAP, mean arterial pressure; PD, pupil dilation; RA, respiration amplitude; SBP, systolic blood pressure; SCL, skin conductance level; SCR, skin conductance response; B, between–subject design study; W, within–subject design study; F, film; I, images; C1, no instruction given (“view”); C2, instruction not to regulate; C3, instruction to maintain target emotion; C4, instruction to respond naturally; C5, a combination of C1–C4; Ntotal, number of participants in the study; Nanalyzed, number of participants in the subsample*.

### Data Extraction

The first author coded the sample sizes, group means, standard deviations, *t* and *p*-values for tests on group effects and participants' mean age of the eligible studies. Another person independently coded 50% of the included studies to evaluate reliability. Correlation analysis confirmed high interrater-reliability (mean *r* = 0.95, range = 0.66–1.0). In addition, inconsistencies between raters were identified and subsequently corrected. Additionally, the psychophysiological measure, and the specific emotion regulation strategy (distraction, reappraisal, suppression, own choice) were coded. When comparing emotion regulation studies, a major problem arises from inconsistencies in the way emotion regulation instructions are labeled. For example, studies that labeled a condition as “suppression” either instructed participants to use reappraisal (Eippert et al., 2007; Bernat et al., 2011) or to suppress thoughts or facial expressions (Gross and Levenson, 1993; Ohira et al., 2006). To prevent confusion, we specifically evaluated the particular emotion regulation instructions as reported in the articles and coded them according to the taxonomy adapted from Webb et al. (2012). See Table 1 for definitions and examples. For this meta-analysis, we also subdivided the control strategies into five types (classifications can be derived from Table 1; adapted from Webb et al., 2012): no instruction at all (i.e., “view”), instruction “not to regulate in a certain manner,” instructions to “respond naturally,” instructions to “maintain” the target emotion or a combination of the above instructions. Furthermore, the researcher(s) also coded whether a study used a between-subject design with two independent groups for the control and the experimental group or a within-subject design with a single group undergoing both regulation and control conditions. In addition the nature of emotion induction if applicable [images, film, music, dyadic interaction, past experience or negative self-belief, threat of shock (ToS), stress task, anger task] was also coded. Finally, we coded the trial duration (i.e., the length of the regulation period of a trial, in seconds). We defined the length of a regulation period as the length of one regulation attempt. In event-related designs a regulation attempt thus corresponds to one trial (i.e., after instruction until picture offset), whereas in studies presenting films or stress tasks, a regulation attempt corresponds to the whole film viewing period or task period (i.e., after instruction until end of film/task).

Regarding electrodermal activity, there was great variability in the quantification of skin conductance across studies. We developed a taxonomy by which we divided electrodermal activity measures in skin conductance level, skin conductance response and number of skin conductance responses (see Table 2). A detailed description of the taxonomy and a table summarizing all included studies on electrodermal responses with information about the categorization can be found in the supplement (p. 2 and Table S2).

### Statistical Analysis

Cohen's *d* was used as the effect size measure in the meta-analyses. For between-subject studies, effect sizes were calculated from the means and standard deviations of the control and experimental (regulation) groups. For within-subject studies, we used the means and standard deviations of the control and experimental (regulation) conditions. If these values were not available, effect sizes were calculated using *t*-values. Furthermore, the variances of the effect sizes were determined. In within-subject designs, the variance of the effect size estimate depends on the correlation between the paired measurements. If the correlation was not available from the original data, the median correlation from the other studies entering the meta-analysis was used. Effect sizes were interpreted based on Cohen's guidelines (Cohen, 1988). Therefore, effects at the 0.2, 0.5, and 0.8 levels were considered as small, medium, and large, respectively.

Since the experimental conditions of the studies differ in many ways, it is unlikely that the studies share a common effect size. Fixed-effect models are therefore implausible. Following recommendations of Borenstein et al. (2010) we conducted random effects meta-analyses. We calculated average effect sizes and 95% confidence intervals (CI). Heterogeneity of effect sizes was assessed with the *I*^2^-statistic which represents the proportion of total variation in the estimated effect sizes that is due to heterogeneity between studies (Higgins and Thompson, 2002). The analyses were performed separated by psychophysiological measure and emotion regulation strategy. Meta-analyses were only conducted when five or more independent samples were available^4^.

For each significant meta-analysis we constructed a funnel plot with the effect sizes on the horizontal axis and their standard errors on the vertical axis. Egger's tests (Egger et al., 1997) were applied to evaluate asymmetry in funnel plots which may be caused by publication bias.

Several studies included two or three assessments within a given measure (e.g., skin conductance level during the regulation of sad and disgusting stimuli) so that there was more than one effect size reported for a specific sample. In these cases, we used the mean of the multiple effect sizes. To calculate the variance of this mean effect size, we assumed that the correlation between the effect sizes was 0.5. If studies reported sufficient results from multiple independent samples (e.g., men and women, prone to disgust vs. not prone to disgust), each of them entered the analysis. Effect sizes for interbeat interval and heart rate were included in the same analyses. To align to polarity of the effect sizes, the parameter for interbeat interval was multiplied by minus one. Thus, a negative size of interbeat interval corresponds to decreased heart rate.

As physiological measures have been shown to discriminate between negative and positive emotional states (Levenson et al., 1990; Bradley and Lang, 2000; Kreibig, 2010), we aimed for distinguishing between positive and negative target emotions in our analyses. Only 13 studies in total (Gross and Levenson, 1997; Demaree et al., 2004; Ohira et al., 2006; Giuliani et al., 2008; Driscoll et al., 2009; Dan-Glauser and Gross, 2011, 2015; Gruber et al., 2014; Baur et al., 2015; Conzelmann et al., 2015; Gomez et al., 2015; Wu et al., 2016; Kotwas et al., 2019) induced positive emotions. Combinations of psychophysiological measure and emotion regulation strategy resulted in a maximum of three studies. Therefore, meta-analyses on the regulation of positive emotions were not computed in the present study. See an overview of studies using positive emotions in the Table S1.

We conducted moderator analyses to test whether features of the experimental context influenced the effect sizes. We used four moderator variables in our analyses: study design (within-subject vs. between-subject), nature of control condition (instruction to respond naturally vs. no instruction), nature of emotion induction (films vs. pictures), and trial duration (i.e., length of a regulation trial, in seconds), as far as there were enough studies for statistical comparison. To evaluate the effects of moderators we used meta-regression analyses and present the regression coefficients.

Statistical analyses were conducted with the metaphor package from R (version 3.2) and SAS 9.4 (SAS Institute Inc., Cary, NC, USA). Statistical significance was defined at the 5% level.

#### Heterogeneity

We investigated whether the variance between the observed effect sizes was larger than what would be expected on the basis of sampling variance alone (Hedges, 1982; Rosenthal and Rubin, 1982). If the effect sizes are heterogeneous it means that the mean effect size does not represent individual effect sizes for studies within the population in that moderators of the effect sizes may be present (e.g., nature of emotion induction). In an analysis with a small number of effect sizes, especially if they are based on small sample size studies, the *Q*-statistic may be non-significant even when there is considerable variability among the effect sizes. Therefore, we computed the percent of variability in effect sizes due to heterogeneity using the *I*^2^ statistic (Higgins and Thompson, 2002). *I* represents the amount of variability in effect sizes that is accounted for by heterogeneity as a proportion of the total variability. According to Higgins and Thompson's (2002) general guidelines, mild heterogeneity would be suggested by an *I*^2^ = 30% of the variability in effect sizes, moderate heterogeneity by an *I*^2^ between 30 and 50%, and notable heterogeneity when *I*^2^ is > 50% of the variability.

#### Moderator Analyses

We conducted moderator analyses to test whether features of the experimental context influenced the observed effect sizes. We used four moderator variables in our analyses: study design (within-subject vs. between-subject), nature of control condition (instruction to respond naturally vs. no instruction)^5^, nature of emotion induction (films vs. pictures)^6^, trial duration (i.e., length of a regulation trial, in seconds), as far as there were sufficient cases for statistical comparison. We used meta-regression (Thompson and Sharp, 1999) to evaluate moderators. The advantage of meta-regression is that continuous moderators (e.g., trial duration) can be evaluated alongside categorical moderators (e.g., within- vs. between-participants designs). For the meta- regressions, β is the beta weight or coefficient assigned to the predictor; *t* (and the associated *p-*value) tests whether the beta weight is significantly different from zero.

## Results

### Descriptive Analyses

**Figure 2:**
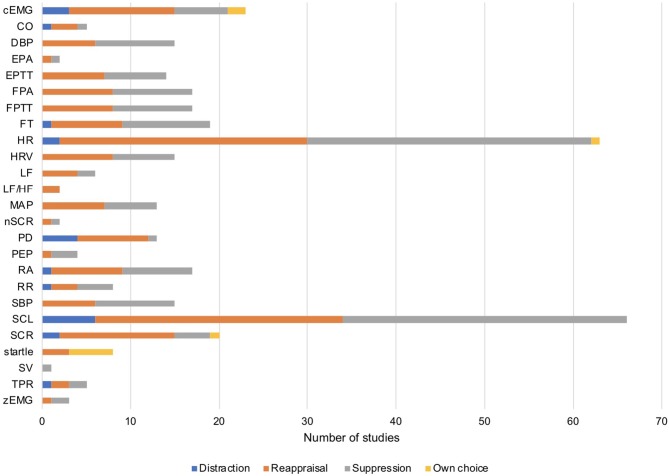
Number of available effect sizes for each measure as a function of emotion regulation strategy (distraction, reappraisal, suppression, own choice). Note that the statistic refers to the k = 78 studies initially identified in our qualitative analysis. cEMG, corrugator activity; CO, cardiac output; DBP, diastolic blood pressure; EPA, ear pulse amplitude; EPTT, ear pulse transit time; FPA, finger pulse amplitude; FPTT, finger pulse transit time; FT, finger temperature; HR, heart rate; HRV, heart rate variability; LF, low frequency HRV; LF/HF, ratio between low and high frequency HRV; MAP, mean arterial pressure; nSCR, number of skin conductance responses; PD, pupil dilation; PEP, pre-ejection period; RA, respiration amplitude; RR, respiration rate; SBP, systolic blood pressure; SCL, skin conductance level; SCR, skin conductance response; SV, stroke volume; TPR, total peripheral resistance; zEMG, zygomatic activity.

Across the 78 studies that were initially considered in our qualitative analysis, heart rate (HR) and skin conductance level (SCL) was measured most frequently, with three times as many effect sizes as for any other measure (see Figure 2 for an overview). Thus, emotion regulation strategies and psychophysiological measures were not evenly represented in the published literature. Certain combinations of emotion regulation strategy and psychophysiological measures occurred frequently in published experiments (e.g., reappraisal and measuring heart rate) whereas other combinations were rare or non-existent (e.g., suppression while measuring stroke volume).

Sixty-nine individual studies entered our quantitative analyses (for a flowchart of the selection and screening process see Figure 1). Study characteristics of these studies are presented in Table 3. There are *n* = 4,474 *unique* individuals across all of the 68 included studies (meaning that this is the total *n* across all studies) with many individuals contributing data to more than one effect size for a total of *n* = 13,380 data points across all meta-analytic comparisons. Because not all studies reported demographic statistics, reported information about age and sex is only an estimated number.

### Meta-Analyses

**Figure 3:**
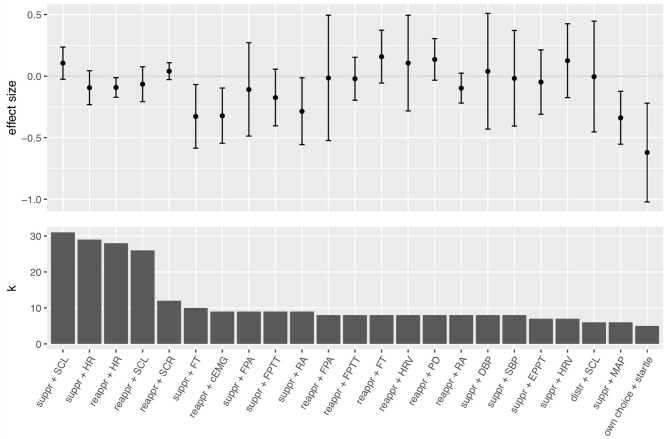
Mean effect sizes and confidence intervals for each conducted meta-analysis (upper panel) sorted by number of samples k of the meta-analysis, respectively (lower panel). Suppr, suppression; reappr, reappraisal; distr, distraction; HR, heart rate; SCL, skin conductance level; SCR, skin conductance response; FT, finger temperature; cEMG, corrugator activity; FPA, finger pulse amplitude; FPTT, finger pulse transit time; RA, respiration amplitude; HRV, heart rate variability; PD, pupil dilation; DBP, diastolic blood pressure; SBP, systolic blood pressure; EPTT, ear pulse transit time; MAP, mean arterial pressure.

As the 68 studies contributed data to multiple effect sizes, we computed 267 individual effect sizes (see Table 3) that entered 24 different meta-analyses (see Table 4 and Figure 3). Overall, computed individual mean effect sizes for each combination of regulation strategy with measure did not exceed d = 0.62 (own choice effect on startle; see Table 4). Figure 3 also highlights that some meta-analyses revealed large confidence intervals and non-significant effect sizes, suggesting that these effects are rather inconsistent (e.g., suppression effect on skin conductance response, ear pulse transit time, diastolic blood pressure and finger pulse amplitude, reappraisal effect on finger pulse amplitude, heart rate variability, and distraction effect on skin conductance level). Largest effect sizes were obtained for electromyographic responses (startle and corrugator activity), followed by suppression effects on some cardiovascular measures (i.e., finger temperature and mean arterial pressure). For many computed mean effect sizes confidence intervals around the mean effect were large (see Figure 3), indicating that the accuracy of our analysis to predict the true effect was rather low. Moreover, heterogeneity differed largely across meta-analyses (see Table 4). For individual forest plots of each meta-analysis see Figures S1–S23.

**Table 4:** Mean computed effect sizes for each emotion regulation strategy and psychophysiological measure.

**Strategy**	**Response system**	**Measure**	**k**	**Effect size**	**SE**	**CI lower**	**CI upper**	***I^**2**^***	***p***	**Direction of effect**
**Distraction**	Electrodermal									
		SCL	6	−0.004	0.175	−0.454	0.447	95.53	0.984	–
**Reappraisal**	Cardiovascular									
		FPA	8	−0.015	0.215	−0.524	0.495	88.90	0.948	–
		FPTT	8	−0.021	0.074	−0.195	0.153	24.73	0.785	–
		FT	8	0.159	0.091	−0.056	0.373	21.99	0.124	–
		HR	28	−0.092	0.039	−0.171	−0.012	21.91	0.026^*^	REG < CTL
		HRV	8	0.106	0.164	−0.282	0.494	87.62	0.537	–
	Electrodermal									
		SCL	26	−0.065	0.069	−0.206	0.077	71.11	0.355	–
		SCR	12	−0.041	0.031	−0.028	0.109	33.01	0.218	–
	Pupillometric									
		PD	8	0.136	0.071	−0.033	0.305	69.82	0.098	–
	Respiratory									
		RA	8	−0.097	0.051	−0.218	0.024	00.00	0.101	–
	Electromyographic									
		cEMG	9	−0.321	0.098	−0.546	−0.096	42.84	0.011^*^	REG < CTL
**Suppression**	Cardiovascular									
		DBP	8	0.039	0.199	−0.431	0.510	83.99	0.849	–
		EPPT	7	−0.048	0.107	−0.309	0.213	54.77	0.670	–
		FPA	9	−0.108	0.165	−0.488	0.272	84.160	0.530	–
		FPTT	9	−0.174	0.100	−0.404	0.057	70.10	0.121	–
		FT	10	−0.327	0.115	−0.586	−0.067	70.03	0.019^*^	REG < CTL
		HR	29	−0.093	0.067	−0.231	0.045	78.28	0.177	–
		HRV	7	0.126	0.122	−0.174	0.425	78.76	0.344	–
		MAP	6	−0.338	0.084	−0.554	−0.123	16.45	0.010^**^	REG < CTL
		RA	9	−0.285	0.118	−0.558	−0.012	61.21	0.042^*^	REG < CTL
		SBP	8	−0.018	0.164	−0.407	0.371	78.32	0.917	–
	Electrodermal									
		SCL	31	0.106	0.064	−0.025	0.236	77.57	0.108	–
**Own choice**	Electromyographic									
		Startle	5	−0.621	0.145	−1.021	−0.219	47.35	0.013^**^	REG < CTL

*k, number of studies; SE, standard error; CI, confidence interval; cEMG, corrugator electromyography; DBP, diastolic blood pressure; EPTT, ear pulse transit time; FPA, finger pulse amplitude; FPTT, finger pulse transit time; FT, finger temperature; HR, heart rate; HRV, heart rate variability; MAP, mean arterial pressure; PD, pupil dilation; RA, respiration amplitude; SBP, systolic blood pressure; SCL, skin conductance level; SCR, skin conductance response. I^2^, percent of variability in effect sizes that is due to heterogeneity between studies*.

* *p ≤ 0.05*,

** *p ≤ 0.01*.

#### Cardiovascular Responses

Reappraisal significantly decreased heart rate (d = −0.09, CI = [−0.17, −0.01], *p* = 0.03, k = 28, *I*^2^ = 21.90), yet the effect size was very small and direction of effects across individual studies were inconsistent (see Figure S6). Reappraisal had no significant effect on all other tested cardiovascular measures (i.e., finger pulse amplitude, finger pulse transit time, finger temperature, and heart rate variability) with mean effect sizes ranging between −0.02 and 0.16 (see Table 4).

Suppression significantly decreased finger temperature (d = −0.33, CI = [−0.59, −0.07], *p* = 0.02, k = 10, *I*^2^ = 70.03; see Figure S16), and mean arterial pressure (d = −0.34, CI = [−0.55, −0.12], *p* = 0.01, k = 6, *I*^2^ = 16.45; see Figure S19), with small to medium effect sizes and mild to notable heterogeneity. Suppression did not significantly change diastolic blood pressure, ear pulse transit time, heart rate, heart rate variability, systolic blood pressure, and skin conductance response (see Table 4 for details and statistics).

#### Electromyographic Responses

When considering studies that instructed participants to choose a strategy that worked best for them only, downregulation of negative emotions had a significant negative effect on the emotion-modulated startle (d = −0.62, CI = [−1.02, −0.22], *p* = 0.01, k = 5, *I*^2^ = 47.35)^7^ with a large effect size and moderate heterogeneity (see Table 4 and Figure S23 for details). This means that the instruction to decrease negative emotions reduced, on average, the startle response compared to the control instruction. Moreover, reappraisal significantly decreased corrugator activity (d = −0.32, CI = [−0.55, −0.10], *p* = 0.01, k = 9, *I*^2^ = 42.84) with medium effect size and moderate heterogeneity (see Table 4 and Figure S2 for details). However, number of studies on the startle (k = 5) and corrugator activity (k = 9) was small and thus should be interpreted with caution.

#### Electrodermal Responses

No significant effect was obtained for distraction on skin conductance level compared to the control condition (d = −0.004, CI = [0.98, 0.45], *p* = 0.45, k = 6, *I*^2^ = 95.35; see Figure S1). Similarly, reappraisal had no significant effect on skin conductance level (d = −0.07, CI = [−0.21, 0.08], *p* = 0.35, k = 26, *I*^2^ = 71.11; see Figure S10) and skin conductance response (d = 0.04, CI = [−0.03, 0.11], *p* = 0.11, k = 12, *I*^2^ = 33.01; see Figure S11), compared to the control condition.

In addition, suppression did not significantly change the skin conductance level (d = 0.11, CI = [−0.03, 0.24], *p* = 0.11, k = 31, *I*^2^ = 77.57; see Table 4 and Figure S22).

#### Respiratory Responses

Suppression significantly decreased respiration amplitude (d = −0.29, CI [−0.56, −0.01], *p* = 0.04, k = 9, *I*^2^ = 61.21; see Figure S20). Sample size was small (k = 9) and thus should be interpreted with caution.

#### Pupillometric Responses

On average, reappraisal did not significantly change pupil dilation in response to negative stimuli compared to a control condition (see Table 4 and Figure S8 for details). Descriptively, this result might have been driven by one study (Bebko et al., 2011) which found a decrease in pupil size during reappraisal, whereas other studies (van Reekum et al., 2007; Urry et al., 2009; Strauss et al., 2016) found an increase in pupil size during reappraisal. Overall sample size (k = 8) was small and thus should be interpreted with caution.

### Evaluation of Publication Bias

For each significant meta-analysis we constructed a funnel plot with the effect sizes on the horizontal axis and their standard errors on the vertical axis. Egger's tests (Egger et al., 1997) were applied to evaluate asymmetry in funnel plots which may be caused by publication bias. Egger's test revealed that there was significant asymmetry only for the effect of reappraisal on heart rate (*p* = 0.008). Individual funnel plots are presented in the supplement (Figure S24).

### Moderator Analyses

We report moderator analyses only for reappraisal and suppression. For distraction and own choice the number of studies was too small or the distributions of the moderators were inadequate.

#### Study Design

Study design (within-subject vs. between-subject) significantly moderated effect sizes of suppression on finger temperature (β = 0.54, *p* ≤ 0.01), finger pulse amplitude (β = 0.78, *p* ≤ 0.001), and heart rate (β = −0.38, *p* ≤ 0.01). See Table 5 for details. The effect of suppression on finger temperature were significant for between-subject design studies (d = −0.62, *p* ≤ 0.001, k = 5), whereas the effect on heart rate became significant for within-subject designs (d = −0.29, *p* ≤ 0.001, k = 10).

**Table 5:** Moderator analyses on study design (within-subject design vs. between-subject design).

**Strategy**	**Measure**	**k**	**k (within)**	**k (between)**	**N total**	**β**	**SE**	***p***
Reappraisal	SCL	26	17	9	1,082	−0.001	0.161	0.997
Reappraisal	HR	28	16	12	1,176	−0.131	0.085	0.134
Suppression	SCL	31	11	20	1,805	−0.176	0.126	0.174
Suppression	FT	10	5	5	701	0.543	0.138	0.004^**^
Suppression	FPTT	9	5	4	608	0.080	0.219	0.725
Suppression	FPA	9	4	5	666	0.775	0.115	0.000^**^
Suppression	RA	9	5	4	467	−0.188	0.263	0.497
Suppression	HR	29	10	19	1,640	−0.379	0.113	0.002^**^

*k, number of studies; SE, standard error; FPA, finger pulse amplitude; FPTT, finger pulse transit time; FT, finger temperature; HR, heart rate; RA, respiration amplitude; SCL, skin conductance level; β regression coefficient (within vs. between)*.

** *p ≤ 0.01*.

#### Nature of Control Instruction

Effect sizes of suppression on finger temperature (β = 0.54, *p* ≤ 0.01), finger pulse transit time (β = 0.42, *p* < 0.05), and finger pulse amplitude (β = 0.78, *p* < 0.001) were significantly moderated by the control instruction (instruction to respond naturally vs. no instruction) (see Table 6). The effect of suppression on heart rate (β = −0.29, *p* < 0.05) and skin conductance level (β = −0.35, *p* ≤ 0.01) was also moderated by the control instruction (instruction to respond naturally vs. no instruction). When studies with no instruction were considered only, suppression significantly increased skin conductance level (d = 0.19, *p* ≤ 0.01, k = 21), decreased finger temperature (d = −0.62, *p* ≤ 0.001, k = 5), and finger pulse transit time (d = −0.40, *p* ≤ 0.01, k = 5). Conversely, when studies with instruction to respond naturally were considered only, suppression significantly decreased heart rate (d = −0.32, *p* ≤ 0.01, k = 8).

**Table 6:** Moderator analyses on nature of control instruction (instruction to respond naturally vs. no instruction).

**Strategy**	**Measure**	**k**	**k C4**	**k C1**	**N total**	**β**	**SE**	***P***
Reappraisal	SCL	23	12	11	986	−0.063	0.127	0.625
	SCR	11	7	4	491	0.087	0.113	0.460
	HR	25	13	12	1,039	−0.033	0.083	0.696
Suppression	SCL	28	7	21	1,665	−0.347	0.130	0.012^*^
	FT	10	5	5	701	0.543	0.138	0.004^*^
	FPTT	9	4	5	608	0.422	0.156	0.030^*^
	FPA	9	4	5	666	0.775	0.115	0.000^**^
	HR	26	8	18	1,500	−0.293	0.130	0.034^*^

*k, number of studies; SE, standard error; FPA, finger pulse amplitude; FPTT, finger pulse transit time; FT, finger temperature; HR, heart rate; SCL, skin conductance level; SCR, skin conductance response; C4, instruction to respond naturally; C1, no instruction; β regression coefficient (respond naturally vs. no instruction)*.

* *p ≤ 0.05*,

** *p ≤ 0.01*.

#### Emotion Induction

Moderator analyses of effect sizes were conducted for film vs. picture only, as too few studies employing other emotion induction methods for each strategy and psychophysiological measure combination were available to interpret moderator analyses in a meaningful way. Emotion induction (films vs. pictures) did not significantly moderate the effect sizes of reappraisal and suppression on skin conductance level and heart rate (see Table 7).

**Table 7:** Moderator analyses on emotion induction (films vs. pictures).

**Strategy**	**Measure**	**k**	**k films**	**k pictures**	**N total**	**β**	**SE**	***p***
Reappraisal	SCL	23	16	7	900	0.126	0.167	0.458
Reappraisal	HR	20	12	8	723	−0.150	0.086	0.101
Suppression	SCL	26	22	4	1,431	0.049	0.187	0.795
Suppression	HR	25	19	6	1,256	0.145	0.144	0.324

*k, number of studies; SE, standard error; HR, heart rate; SCL, skin conductance level; β, regression coefficient (films vs. pictures)*.

#### Trial Duration

Trial duration significantly moderated the effect of reappraisal on skin conductance response (β = −0.03, *p* = 0.05, k = 12) and the effect of suppression on skin conductance level (β = −0.03, *p* < 0.05, k = 31), diastolic (β = −0.41, *p* < 0.05, k = 8) and systolic blood pressure (β = −0.39, *p* < 0.01, k = 8) in that the effect became more negative with longer trial durations (see Table 8). The moderating effect of trial duration on suppression and skin conductance level was mainly driven by one study (Yuan et al., 2014).

**Table 8:** Moderator analyses on trial duration.

**Strategy**	**Measure**	**k**	**N total**	**β**	**SE**	***p***
Distraction	SCL	6	287	0.084	0.081	0.354
Reappraisal	HR	28	1176	0.015	0.012	0.209
	HRV	8	305	0.071	0.053	0.232
	PD	8	250	−2.492	1.996	0.258
	SCL	26	1,082	0.021	0.021	0.324
	SCR	12	530	−0.028	0.013	0.053^*^
	cEMG	9	354	0.000	0.051	0.997
Suppression	DBP	8	440	−0.408	0.141	0.028^*^
	EPPT	7	551	0.022	0.024	0.403
	FPA	9	666	−0.030	0.039	0.464
	FPTT	9	608	−0.011	0.026	0.677
	FT	10	701	0.130	0.086	0.172
	HR	29	1640	0.028	0.022	0.214
	HRV	7	491	0.044	0.047	0.392
	RA	9	467	−0.032	0.048	0.526
	SBP	8	440	−0.387	0.094	0.006^**^
	SCL	31	1,805	−0.026	0.012	0.039^*^

*k, number of studies; SE, standard error; cEMG, corrugator electromyography; DBP, diastolic blood pressure; EPTT, ear pulse transit time; FPA, finger pulse amplitude; FPTT, finger pulse transit time; FT, finger temperature; HR, heart rate; HRV, heart rate variability; MAP, mean arterial pressure; PD, pupil dilation; RA, respiration amplitude; SBP, systolic blood pressure; SCL, skin conductance level; SCR, skin conductance response; β regression coefficient (refers to 1 min change in trial duration)*.

* *p ≤ 0.05*,

** *p ≤ 0.01*.

## Discussion

Over the past two decades, emotion regulation has become a vibrant research field. Our literature search corroborates this trend. It revealed an increase of almost 60% of potentially relevant publications for our meta-analysis within the recent 3 years. The vast growth of literature illustrates a vigorous interest in understanding the psychophysiological mechanisms of emotion regulation.

Previous studies on the psychophysiological responses to emotion regulation revealed inconsistent results. Moreover, distraction and reappraisal strategies appeared to have no or little effect on psychophysiology (Webb et al., 2012), and suppression significantly increased sympathetic arousal (Gross and Levenson, 1993; Gross, 1998a). This meta-analysis provides the first attempt to elucidate common trends with means of a quantitative summary of the effects of common emotion regulation strategies on different cardiovascular, electrodermal, respiratory, pupillometric, and electromyographic measures. We performed a structured literature review and conducted a meta-analysis for each combination of psychophysiological measure and emotion regulation strategy whenever there were enough studies available. In brief, we found that suppression significantly decreased mean arterial pressure, finger temperature, and respiration amplitude, whereas reappraisal led to decreased heart rate and decreased corrugator activity (see Table 4 and Figure 3 for an overview of effects). When participants were free to choose between emotion regulation strategies, a significant inhibition of the emotion-modulated startle (sometimes referred to as fear-potentiated startle) response could be observed. Due to the limited number of studies on distraction, we were not able to conduct meta-analyses on psychophysiological responses except for skin conductance level, and this meta-analysis revealed no significant effect. Publication bias appeared to have an overall minor effect.

As Figure 3 illustrates, aggregated effect sizes from the tested autonomic responses were small in general. We did not compute an overall effect size across all psychophysiological measures. Yet aggregated effect sizes for each psychophysiological measure correspond with the results reported by Webb et al.'s meta-analysis (Webb et al., 2012). They had reported an overall small negative effect of response modulation (e.g., suppression strategies) on psychophysiology (*d* = 0.19, [CI = 0.14, 0.01]). Attentional deployment (e.g., distraction strategies) had no significant effect on physiological measures (*d* = 0.00, CI = [0.14, 0.15]), and so did cognitive change (e.g., reappraisal) (*d* = 0.05, [CI = 0.07 to 0.16]) (Webb et al., 2012). We conclude that effects of emotion regulation on autonomic measures—if at all present—seem to be rather small and raise the question whether emotion regulation success can be reliably quantified with autonomic measures. It should however be noted that the psychophysiological measures entering our analysis were limited. Figure 2 illustrates that there were a number of measures not included as too few studies were available. For example, measures of cardiac function that can be derived via impedance cardiography have received scant attention in the previous literature but provide promising results: Studies have shown that emotion regulation changed total peripheral resistance with medium to large effect sizes (Jamieson et al., 2012, 2013; Peters et al., 2014; Peters and Jamieson, 2016).

Activation of the sympathetic nervous system causes an increase in skin conductivity, pupil dilation, heart rate, pre-ejection period, blood pressure, peripheral vasoconstriction, and increased respiration amplitude and respiration rate. Successful emotion regulation should be accompanied by a reduction of sympathetic activity (McRae and Shiota, 2017). Our study reveals that the effects are not quite that straightforward. Suppression lowered finger temperature (indicative of increased sympathetic activity), yet also decreased mean arterial pressure and respiration amplitude (indicative of lower sympathetic activity). Similarly, reappraisal decreased heart rate (indicative of lower sympathetic activity) but did not change any of the other tested autonomic measures. McRae and Shiota (2017) point out that psychophysiological effects often diverge in patterns that correspond to different psychological states (Kreibig, 2010; Shiota et al., 2011), which can result in misinterpretations about the association between psychophysiological responses and the underlying psychological processes (Cacioppo and Tassinary, 1990; Cacioppo et al., 2007). Psychophysiological responses are usually influenced by various factors, such as stress, workload, or tiredness, and thus may distort the effects of emotion regulation. Decreased pupil size during reappraisal was observed in one study and has been interpreted to be the result of decreased emotional arousal (Bebko et al., 2011). Alternatively, studies have interpreted larger pupil size during reappraisal as an indicator of higher cognitive effort (Urry et al., 2006; van Reekum et al., 2007). They infer that pupil size may increase during successful emotion regulation as an indicator of increased cognitive processing. The ambiguity of such effects implies that we need a better understanding of cognitive and emotional processes causing autonomic change, and how these changes relate to emotion regulation success.

Another problem is the inconsistency of direction of effect sizes. Different directions of effect sizes rendered the meta-analyses insignificant and infer that there are important factors not yet understood. For example, the meta-analysis of pupil dilation during reappraisal (see Figure S8) revealed that one study (Bebko et al., 2011), which received a strong weight in the analysis, found a significant decrease in pupil diameter during reappraisal, while other studies found an increase in pupil diameter (e.g., van Reekum et al., 2007; Urry et al., 2009; Strauss et al., 2016). Similarly, our meta-analysis on heart rate during suppression (see Figure S17) revealed that studies found mean heart rate acceleration in response to suppression (e.g., Hagemann et al., 2006; Stiller et al., 2019), whereas other studies found a heart rate deceleration (Kunzmann et al., 2005; Dan-Glauser and Gross, 2011, 2015). Therefore, the second aim of the present work was to explore the impact of methodological differences using several moderators (trial duration, nature of emotion induction, nature of control instruction, study design).

Effects of suppression on heart rate, finger temperature and finger pulse amplitude were significantly moderated by study design (within vs. between-subject). Between-subject design studies showed a significant decrease in finger temperature and finger pulse amplitude during suppression whereas studies with a within-subject design revealed no significant effect. Conversely, within-subject design studies showed a significant decrease in heart rate whereas studies with a between-subject design revealed no significant effect. The moderating effect of study design on heart rate might also reflect that between-subject design studies in this particular meta-analysis assessed extremely diverse emotion induction methods. For example, two studies (Butler et al., 2006; Ben-Naim et al., 2013) assessed emotion regulation in dyadic interactions. Hagemann et al. (2006) used startle tones in combination with pictures. Rohrmann et al. (2009), Gross (1998a), Denson et al. (2011) used film stimuli. Within-subject design studies considered in this meta-analysis used films and pictures only. Therefore, the nature of emotion induction may account for some variance in the effect sizes obtained across studies using between-subject designs. When data from more studies will be available in the future, it might be possible to confirm this assumption.

Effects of reappraisal and suppression on several electrodermal and cardiovascular measures (i.e., skin conductance level, finger temperature, finger pulse transit time, finger pulse amplitude, and heart rate) were significantly moderated by the nature of control instructions. Except for finger pulse amplitude, the effects became significant when no instruction (i.e., “view” instruction) was given but did not become significant when the instruction to respond naturally was given. This does not correspond with findings by Webb et al. (2012) who found that emotion regulation strategies in general had smaller effects on experiential, behavioral and physiological measures combined when the control condition required participants to “view” or “not to regulate” and larger effects when the control condition required participants to respond naturally. In contrast to our study, they did not determine the moderating effect of control instruction on physiological effects of emotion regulation but considered the overall effect of psychophysiological, behavioral and experiential measures. Control conditions requiring participants to simply view a negative stimulus might correspond to a physiological baseline condition. However, when receiving the instruction to respond naturally, participants might unconsciously pay more attention to their emotional response, which may be particularly sensitive to psychophysiological responses.

Trial duration significantly moderated effect sizes of suppression on skin conductance level, diastolic and systolic blood pressure, and of reappraisal on skin conductance response in that the effects became more negative with increasing trial length. Studies on electrodermal responses may be difficult to compare within the conducted meta-analyses because trial durations varies largely across studies. This might be especially problematic for skin conductance level, as longer time windows carry the risk that non-specific skin conductance responses occur. If these phasic responses are not separated from the tonic parts, they might influence the absolute skin conductance level (Boucsein et al., 2012). Hence, skin conductance level assessed over several seconds in an event-related design might be different than skin conductance level assessed over several minutes in a block-design. We accounted for this variability in parts by conducting a moderator analysis with trial duration as the moderator. We observed effects in both positive and negative direction. Studies with very short trial duration tend to report an increase in skin conductance, whereas studies with longer or extremely long trial durations tend to report a decrease in skin conductance. However, we acknowledge that our analysis did not allow to differentiate for example between studies that assessed skin conductance averages but eliminated the tonic parts (Hallam et al., 2015; Plieger et al., 2017) and studies that assessed skin conductance level without separating the phasic from the tonic responses. We encourage future researcher to use similar research methodology and terminology as suggested by the committee report on publication recommendations (Boucsein et al., 2012) to make studies more comparable in the future. In total, the varying effects of skin conductance across studies may be in part due to the high variability in assessment and quantification.

Compared to the tested *autonomic* responses (i.e., cardiovascular, electrodermal, pupillometric and respiratory responses), our present analysis revealed that effects of measures assessed with *electromyography* were medium and consistent across individual studies (see Figures S2, S23). Regarding the emotion-modulated startle, we found a significant decrease through emotion downregulation with a mean effect size of d = −0.62. Corrugator activity significantly decreased with reappraisal of negative emotions with a medium effect size of d = −0.32. As both analyses included a rather small number of studies resulting in large confidence intervals, they should be treated with caution (see Figure 3). Nevertheless, the results on electromyography showed more consistent results compared to the autonomic measures assessed in the present review and this encourages possible reasons that might have accounted for this consistency.

Studies have shown that both the emotion-modulated startle and corrugator activity are specific to valence: The startle is inhibited in response to pleasant but potentiated in response to unpleasant stimuli with stronger responses for high- than for low-arousing stimuli (Vrana et al., 1988; Bradley et al., 1993a; Hamm et al., 1997; Schupp et al., 1997; Hawk and Cook, 2000). Corrugator supercilii is generally considered to correspond to changes in valence, too (Tassinary et al., 2007). The valence-specificity might facilitate to measure the correspondence to changes in valence and hence allows to track the regulation effect more closely, compared with autonomic measures that rather reflect changes in arousal. However, there are also studies showing that in the context of emotion regulation, the startle response is more sensitive to changes in arousal (Dillon and LaBar, 2005; Zaehringer et al., 2018).

Animal studies have shown that the amygdala, a key structure in emotion processing, directly modulates the auditory startle reflex via modulation of midbrain neurons (Rosen and Davis, 1988; Davis, 1992), which has been recently complemented by fMRI work in human subjects (Kuhn et al., 2019). Researcher have argued that the emotional modulation as indexed by the startle reflex may serve as a direct indicator of amygdala activation independent of task demands (Grillon and Baas, 2003). Similarly, the amygdala projects to the facial motor nucleus thereby coupling emotional facial expressions to the motive circuit (Davis, 2000). The amygdala is a robust neural target of emotion regulation (Buhle et al., 2014) and altered amygdala activation with emotion regulation thus likely mediates the modulatory effect on the startle response and corrugator activity. Taken together, the specificity to the valence dimension and the direct modulation via the brain's motivational system may contribute to the findings of emotion regulation effects on emotion modulated startle and corrugator activity.

With regard to the emotion-modulated startle, it is also possible that the emotion regulation instruction might have influenced the obtained effect sizes. Participants in these studies were free to choose an emotion regulation strategy that worked best for them. By allowing participants to choose from different strategies, they might be more successful in regulating their emotions, which could result in larger effects. Moreover, the startle response unfolds within milliseconds, whereas autonomic responses such as pupil dilation, electrodermal responses, and heart rate variability rather unfold over several seconds, or even minutes. Therefore, the startle response may be easier to measure because it is clearly time-locked to the startle probe and all changes can be measured in studies with shorter observation times during the trials, whereas a skin conductance response with a slower response latency to peak may carry over effects to the next trial. In addition, emotion-modulated startle studies largely converge on the measurement and quantification of the startle response, whose setup is known to be relatively simple. In our meta-analysis on the emotion-modulated startle, all studies rectified and integrated the raw EMG signal with a time constant of 20 ms, calculated the startle amplitude by subtracting a 20 or 50 ms pre-startle baseline from the peak 20–120 or 20–150 ms after startle probe onset and finally t- or z-transformed the mean amplitudes (Jackson et al., 2000; Dillon and LaBar, 2005; Golkar et al., 2014; Conzelmann et al., 2015).

In contrast, we observed tremendous variation in the quantification of the autonomic indices. For example, studies on skin conductance level during reappraisal assessed baseline activity during a neutral condition that included the presentation of neutral stimuli (Wolgast et al., 2011; Lohani and Isaacowitz, 2014), right before stimulus onset (e.g., Shiota and Levenson, 2009), right before instruction (Opitz et al., 2014), after instruction (Urry et al., 2009), or reported no baseline assessment (Goldin et al., 2019). These studies then either subtracted mean activity of the respective baseline from mean activity during the regulation period (e.g., Shiota and Levenson, 2009; Opitz et al., 2014), calculated raw means (Goldin et al., 2019), or area under the curve (Urry et al., 2009). It should be noted that these observations remain solely on a descriptive level. We did not conduct a moderator analysis to account for this variation since too few studies were available. Future studies would be helpful to corroborate our considerations.

The meta-analyses we presented in this article suggest that electromyographic measures such as the emotion-modulated startle might be robust options to assess emotion regulation effects, whereas autonomic measures might be context dependent and thus should be selected carefully. Autonomic measures are still important and interesting for emotion regulation research as they allow to track the extended reaction of the body to an emotional event or a series of events, whereas the emotion-modulated startle is being assessed at one given time and thus does not allow to track the time-course of the regulation period.

### Limitations and Future Research

While the present study represents the first meta-analysis of specific psychophysiological effects during distraction, reappraisal, suppression, and instructions to choose a downregulation strategy, it is not without limitations. First of all, we emphasize that the number of available studies was small with the exception of heart rate and skin conductance level. In particular, most of the significant meta-analyses in the present study included few studies and these studies often stemmed from an even smaller number of labs (e.g., mean arterial pressure, finger temperature; see Figure 3). Thus, we need more research to test whether the effects would become insignificant with increasing number of independent studies. Similarly, absence of significance in meta-analyses with small number of samples should not be taken as evidence that there is no effect at all. Thus, studies that assess less common psychophysiological measures and emotion regulation instructions are urgently needed to increase knowledge about psychophysiological responses during emotion regulation.

Furthermore, no meta-analysis is free of a potential publication bias. The bias refers to the phenomenon that significant findings get published earlier and are more likely than non-significant findings. Statistical analyses indicated that there might be some publication bias, but this seemed not to appreciably impact the results. In addition, psychophysiological measures are usually not the primary outcome of emotion regulation studies, and many published studies have reported negative findings. Thus, we consider the publication bias to be relatively small in this review.

We also highlight the substantial variability in the research methodology used across the emotion regulation studies included in our meta-analysis. We explored the impact of methodological differences using several moderators (trial duration, nature of emotion induction, nature of control instruction, study design) and showed that central design aspects are explaining some differences in the overserved autonomic effect sizes. This raises the question to which degree the studies included in the present review are actually comparable.

Sample size was very small and conducting the meta-analyses and moderator analyses required a large number of separate analyses. In light of this, significant results presented here should be treated with caution as multiple comparisons might have increased the chances of false discovery. More research is needed to confirm our results. We also acknowledge that we assessed a limited sample of potential moderators. As mentioned above, there was tremendous variation in the quantification of the autonomic indices, which we were not able to account for as there were too few studies available to conduct meaningful moderator analyses. Finally, we highlight that our meta-analysis was limited to the regulation of negative emotions only, mainly focusing on reappraisal and suppression.

In light of these limitations, we need particularly larger and more comparable studies with identical setup to control the moderator variables identified in this meta-analysis (in particular trial duration, comparable control conditions and the same study design). One important future direction for researchers in the area of psychophysiological response patterns to emotion regulation is to design large-scale, comprehensive studies that directly compare psychophysiological measures and emotion regulation strategies ideally using the same assessment and quantification of psychophysiological responses.

With psychophysiological recordings we cannot control which regulation strategies are really being applied by participants. The variability of autonomic responding across different emotion regulation contexts further complicates an accurate interpretation of effects and may be particularly problematic in studies focusing on just one psychophysiological outcome measure. Experiments using simultaneous recordings from multiple psychophysiological channels would be helpful to e.g., identify potential response patterns uniquely characterizing different emotion regulation strategies (e.g., pupil, heart rate, skin conductance, etc.). However, major progress is unlikely without coordinated effort across labs to systematically address these questions.

There is also a need for studies that carefully tease apart attention, arousal and other cognitive processes that may influence autonomic responses in order to gain a better understanding of the interpretation of autonomic responses during emotion regulation. Systematic variations in different experimental setups may help to dissociate the underlying cognitive and emotional processes that cause autonomic activity in order to draw clear inferences.

## Conclusion

This meta-analysis represents the first attempt to determine the mean effects of different emotion regulation strategies on individual psychophysiological measures. Our results indicate that (a) effects of reappraisal decreased heart rate and corrugator activity, whereas suppression increased sympathetic arousal but decreased respiration amplitude and mean arterial pressure, (b) effects of autonomic measures, even if significant, were small and heterogeneous across studies, while electromyographic measures showed medium effect sizes and (c) the study design, control instruction and trial duration moderated some but not all effect sizes. As available studies were few, our findings remain preliminary. In order to use meta-analyses to compare effects of psychophysiological responses in different regulation contexts, more comparable methodological set-ups should be used in the empirical study of emotion regulation. The induction of specific types of emotions and the assessment of less common psychophysiological measures and regulation strategies will allow future meta-analyses to fully discover the potential influences on psychophysiological response during emotion regulation.

## Data Availability Statement

All datasets generated for this study are included in the article/Supplementary Material.

## Author Contributions

JZ, CP, CS, and GE were involved in the conception of the work. JZ planned and conducted the literature search, coded the data and together with CJ-S designed and carried out the data analysis. JZ drafted the manuscript. CP, CJ-S, CS, and GE revised it critically for important intellectual content.

### Conflict of Interest

The authors declare that the research was conducted in the absence of any commercial or financial relationships that could be construed as a potential conflict of interest.

## Footnotes

^1^Acceptance has become increasingly popular across the emotion regulation literature too, yet there has been a debate as to whether it belongs to antecedent (Webb et al., 2012) or response-focused processes (Hofmann and Asmundson, 2008) and as to whether it is a strategy or rather a function of different strategies. Given that very few studies on acceptance assessed psychophysiological responses, it is not included in the present review.

^2^It should be noted that there might be more aspects of visual stimuli that could possibly influence effect sizes. For example, within the field of visual perception, studies show that faces are not as evocative as scenes (Alpers et al., 2011; Wangelin et al., 2012). A fine-grained moderator analysis of different aspects of picture and film stimuli however was not possible due to the small number of studies available and because most studies included in the present analysis used a blend of negative scenes and faces as stimuli.

^3^The search process was updated two times in total. The first search yielded a total of 848 potentially relevant articles on January 22, 2016 (after duplicates were removed). A second search 1 year later (on February 8, 2017) yielded an additional 210 potentially relevant articles (after duplicates were removed). A third search 2 years later (on July 18, 2019) yielded an additional 295 potentially relevant articles (after duplicates were removed).

^4^Some studies included several independent samples. The minimum number of independent studies required to conduct a meta-analysis was accepted as three.

^5^We were unable to test other types of control instructions as there were too few studies available.

^6^We were unable to test other types of emotion inductions (i.e., music, dyadic interaction, past experience or personally relevant thought, threat of shock, stressor task, anger task) as there were too few studies available.

^7^Instructions to downregulate negative emotions (own choice and reappraisal instructions combined) had a significant negative effect on the emotion-modulated startle too (d = −0.44, CI = [−0.75, −0.14], *p* = 0.01, k = 8, *I*^2^ = 74.76).
