# Supplementary material for: Psychophysiological Effects of Downregulating Negative Emotions: Insights From a Meta-Analysis of Healthy Adults
Source: Front Psychol. 2020 Apr 16;11:470. doi: 10.3389/fpsyg.2020.00470 (PMC7177019; doi:10.3389/fpsyg.2020.00470)
Supplement: Supplementary file 1 [file Data_Sheet_1.docx]

**Supplement**

Psychophysiological effects of downregulating negative emotions: Insights from a meta-analysis of healthy adults

Jenny Zaehringer*^1,2^, Christine Jennen-Steinmetz^3^, Christian Schmahl,^1 4^ Gabriele Ende^2^ & Christian Paret*^1 5^

^1^ Department of Psychosomatic Medicine and Psychotherapy, Central Institute of Mental Health Mannheim, Medical Faculty Mannheim/Heidelberg University, Germany

^2^ Department Neuroimaging, Central Institute of Mental Health Mannheim, Medical Faculty Mannheim/Heidelberg University, Germany

^3^ Department of Biostatistics, Central Institute of Mental Health Mannheim, Medical Faculty Mannheim/Heidelberg University, Germany

4 Department of Psychiatry, Schulich School of Medicine and Dentistry, Western University, London, Ontario, Canada

5 Sagol Brain Institute, Wohl Institute for Advanced Imaging, Tel-Aviv Sourasky Medical Centre, Tel-Aviv, Israel

**Electrodermal responses**

Boucsein et al. (2012) define phasic electrodermal responses as short-lasting changes in electrodermal activity most commonly reported as a peak amplitude (including non-zero responses) or magnitude (including only responses above a defined minimum) after a specific stimulus. Phasic skin conductance responses are thus only a small fraction of the skin conductance level. Tonic skin conductance level refers to the slowly changing level of skin conductance “typically computed as a mean of several measurements taken during a specific time period.” (Boucsein et al., 2012, p. 1026). In addition, non-specific skin conductance responses may be assessed during periods of nonstimulation and they are commonly computed as number of skin conductance responses per unit of time (nSCR). The inspection of included studies in the present review however showed that the distinction between short-lasting changes and slow-changing level in skin conductance are rather fluent in emotion regulation studies. In the studies included in our analyses, skin conductance levels (i.e. average values) were assessed over trials lasting between 4 and 590s seconds, whereas skin conductance responses were assessed over trial durations lasting between 5 and 1800 seconds. To overcome this problem, we created a taxonomy to divide skin conductance level and skin conductance response with definitions adapted to the emotion regulation literature. The taxonomy and a full list of studies with detailed explanation why we categorized them as either “skin conductance response” or “skin conductance level” can be found in the Table S2. In brief, we distinguished between indices reflecting the maximum amplitude and indices reflecting the duration of the skin conductance signal (i.e. averages). In particular, we defined skin conductance responses as maximum amplitude, magnitude, or peak occurring in a particular time after stimulus onset. These amplitudes may be baseline corrected and averaged over time. Non-specific skin conductance responses occurring during longer periods of time were also regarded as “skin conductance responses”, if they were reported as maximum amplitudes. We defined skin conductance level as the mean skin conductance over a specific period of time that can range between several seconds and several minutes. If skin conductance was calculated as the area under the curve or the integrated signal over a period of time, we also defined these measures as skin conductance level, since they are also affected by the duration of the response.

**Figures**

**Distraction strategies**

**

*Figure S1*. Statistics and results from the meta-analysis on skin conductance level (SCL) during distraction. Each row represents one sample. Middle column: ‘effect size’ refers to effect size of the sample. ‘CI’ refers to the 95% confidence interval. The right column shows a forest plot with effect sizes and 95% confidence intervals (CI). The diamond size of the individual effect sizes of each sample refers to the relative weight of the effect.

**Reappraisal strategies**

**

*Figure S2.* Statistics and results from the meta-analysis on corrugator activity (cEMG) during reappraisal. Each row represents one sample. Middle column: ‘effect size’ refers to effect size of the sample. ‘CI’ refers to the 95% confidence interval. The right column shows a forest plot with effect sizes and 95% confidence intervals (CI). The diamond size of the individual effect sizes of each sample refers to the relative weight of the effect.

**

*Figure S3.* Statistics and results from the meta-analysis on finger pulse amplitude (FPA) during reappraisal. Each row represents one sample. Middle column: ‘effect size’ refers to effect size of the sample. ‘CI’ refers to the 95% confidence interval. The right column shows a forest plot with effect sizes and 95% confidence intervals (CI). The diamond size of the individual effect sizes of each sample refers to the relative weight of the effect.

**

*Figure S4.* Statistics and results from the meta-analysis on finger pulse transit time (FPTT) during reappraisal. Each row represents one sample. Middle column: ‘effect size’ refers to effect size of the sample. ‘CI’ refers to the 95% confidence interval. The right column shows a forest plot with effect sizes and 95% confidence intervals (CI). The diamond size of the individual effect sizes of each sample refers to the relative weight of the effect.

**

*Figure S5.* Statistics and results from the meta-analysis on finger temperature (FT) during reappraisal. Each row represents one sample. Middle column: ‘effect size’ refers to effect size of the sample. ‘CI’ refers to the 95% confidence interval. The right column shows a forest plot with effect sizes and 95% confidence intervals (CI). The diamond size of the individual effect sizes of each sample refers to the relative weight of the effect.

**

*Figure S6.* Statistics and results from the meta-analysis on heart rate (HR) during reappraisal. Each row represents one sample. Middle column: ‘effect size’ refers to effect size of the sample. ‘CI’ refers to the 95% confidence interval. The right column shows a forest plot with effect sizes and 95% confidence intervals (CI). The diamond size of the individual effect sizes of each sample refers to the relative weight of the effect.

**

*Figure S7.* Statistics and results from the meta-analysis on heart rate variability (HRV) during reappraisal. Each row represents one sample. Middle column: ‘effect size’ refers to effect size of the sample. ‘CI’ refers to the 95% confidence interval. The right column shows a forest plot with effect sizes and 95% confidence intervals (CI). The diamond size of the individual effect sizes of each sample refers to the relative weight of the effect.

**

*Figure S8.* Statistics and results from the meta-analysis on pupil dilation (PD) during reappraisal. Each row represents one sample. Middle column: ‘effect size’ refers to effect size of the sample. ‘CI’ refers to the 95% confidence interval. The right column shows a forest plot with effect sizes and 95% confidence intervals (CI). The diamond size of the individual effect sizes of each sample refers to the relative weight of the effect.

**

*Figure S9.* Statistics and results from the meta-analysis on respiration amplitude (RA) during reappraisal. Each row represents one sample. Middle column: ‘effect size’ refers to effect size of the sample. ‘CI’ refers to the 95% confidence interval. The right column shows a forest plot with effect sizes and 95% confidence intervals (CI). The diamond size of the individual effect sizes of each sample refers to the relative weight of the effect.

**

*Figure S10.* Statistics and results from the meta-analysis on skin conductance level (SCL) during reappraisal. Each row represents one sample. Middle column: ‘effect size’ refers to effect size of the sample. ‘CI’ refers to the 95% confidence interval. The right column shows a forest plot with effect sizes and 95% confidence intervals (CI). The diamond size of the individual effect sizes of each sample refers to the relative weight of the effect.

**

*Figure S11.* Statistics and results from the meta-analysis on skin conductance response (SCR) during reappraisal. Each row represents one sample. Middle column: ‘effect size’ refers to effect size of the sample. ‘CI’ refers to the 95% confidence interval. The right column shows a forest plot with effect sizes and 95% confidence intervals (CI). The diamond size of the individual effect sizes of each sample refers to the relative weight of the effect.

**Suppression strategies**

**

*Figure S12.* Statistics and results from the meta-analysis on diastolic blood pressure (DBP) during suppression. Each row represents one sample. Middle column: ‘effect size’ refers to effect size of the sample. ‘CI’ refers to the 95% confidence interval. The right column shows a forest plot with effect sizes and 95% confidence intervals (CI). The diamond size of the individual effect sizes of each sample refers to the relative weight of the effect.

**

*Figure S13. S*tatistics and results from the meta-analysis on ear pulse transit time (EPTT) during suppression. Each row represents one sample. Middle column: ‘effect size’ refers to effect size of the sample. ‘CI’ refers to the 95% confidence interval. The right column shows a forest plot with effect sizes and 95% confidence intervals (CI). The diamond size of the individual effect sizes of each sample refers to the relative weight of the effect.

**

*Figure S14.* Statistics and results from the meta-analysis on finger pulse amplitude (FPA) during suppression. Each row represents one sample. Middle column: ‘effect size’ refers to effect size of the sample. ‘CI’ refers to the 95% confidence interval. The right column shows a forest plot with effect sizes and 95% confidence intervals (CI). The diamond size of the individual effect sizes of each sample refers to the relative weight of the effect.

**

*Figure S15.* Statistics and results from the meta-analysis on finger pulse transit time (FPTT) during suppression. Each row represents one sample. Middle column: ‘effect size’ refers to effect size of the sample. ‘CI’ refers to the 95% confidence interval. The right column shows a forest plot with effect sizes and 95% confidence intervals (CI). The diamond size of the individual effect sizes of each sample refers to the relative weight of the effect.

**

*Figure S16.* Statistics and results from the meta-analysis on finger temperature (FT) during suppression. Each row represents one sample. Middle column: ‘effect size’ refers to effect size of the sample. ‘CI’ refers to the 95% confidence interval. The right column shows a forest plot with effect sizes and 95% confidence intervals (CI). The diamond size of the individual effect sizes of each sample refers to the relative weight of the effect.

**

*Figure S17.* Statistics and results from the meta-analysis on heart rate (HR) during suppression. Each row represents one sample. Middle column: ‘effect size’ refers to effect size of the sample. ‘CI’ refers to the 95% confidence interval. The right column shows a forest plot with effect sizes and 95% confidence intervals (CI). The diamond size of the individual effect sizes of each sample refers to the relative weight of the effect.

**

*Figure S18.* Statistics and results from the meta-analysis on heart rate variability (HRV) during suppression. Each row represents one sample. Middle column: ‘effect size’ refers to effect size of the sample. ‘CI’ refers to the 95% confidence interval. The right column shows a forest plot with effect sizes and 95% confidence intervals (CI). The diamond size of the individual effect sizes of each sample refers to the relative weight of the effect.

**

*Figure S19.* Statistics and results from the meta-analysis on mean arterial pressure (MAP) during suppression. Each row represents one sample. Middle column: ‘effect size’ refers to effect size of the sample. ‘CI’ refers to the 95% confidence interval. The right column shows a forest plot with effect sizes and 95% confidence intervals (CI). The diamond size of the individual effect sizes of each sample refers to the relative weight of the effect.

**

*Figure S20.* Statistics and results from the meta-analysis on respiration amplitude (RA) during suppression. Each row represents one sample. Middle column: ‘effect size’ refers to effect size of the sample. ‘CI’ refers to the 95% confidence interval. The right column shows a forest plot with effect sizes and 95% confidence intervals (CI). The diamond size of the individual effect sizes of each sample refers to the relative weight of the effect.

**

*Figure S21.* Statistics and results from the meta-analysis on systolic blood pressure (SBP) during suppression. Each row represents one sample. Middle column: ‘effect size’ refers to effect size of the sample. ‘CI’ refers to the 95% confidence interval. The right column shows a forest plot with effect sizes and 95% confidence intervals (CI). The diamond size of the individual effect sizes of each sample refers to the relative weight of the effect.

**

*Figure S22.* Statistics and results from the meta-analysis on skin conductance level (SCL) during suppression. Each row represents one sample. Middle column: ‘effect size’ refers to effect size of the sample. ‘CI’ refers to the 95% confidence interval. The right column shows a forest plot with effect sizes and 95% confidence intervals (CI). The diamond size of the individual effect sizes of each sample refers to the relative weight of the effect.

**Own choice strategies**

**

*Figure S23.* Statistics and results from the meta-analysis on emotion-modulated startle during downregulation with own choice. Each row represents one sample. Middle column: ‘effect size’ refers to effect size of the sample. ‘CI’ refers to the 95% confidence interval. The right column shows a forest plot with effect sizes and 95% confidence intervals (CI). The diamond size of the individual effect sizes of each sample refers to the relative weight of the effect.

**Funnel Plots**


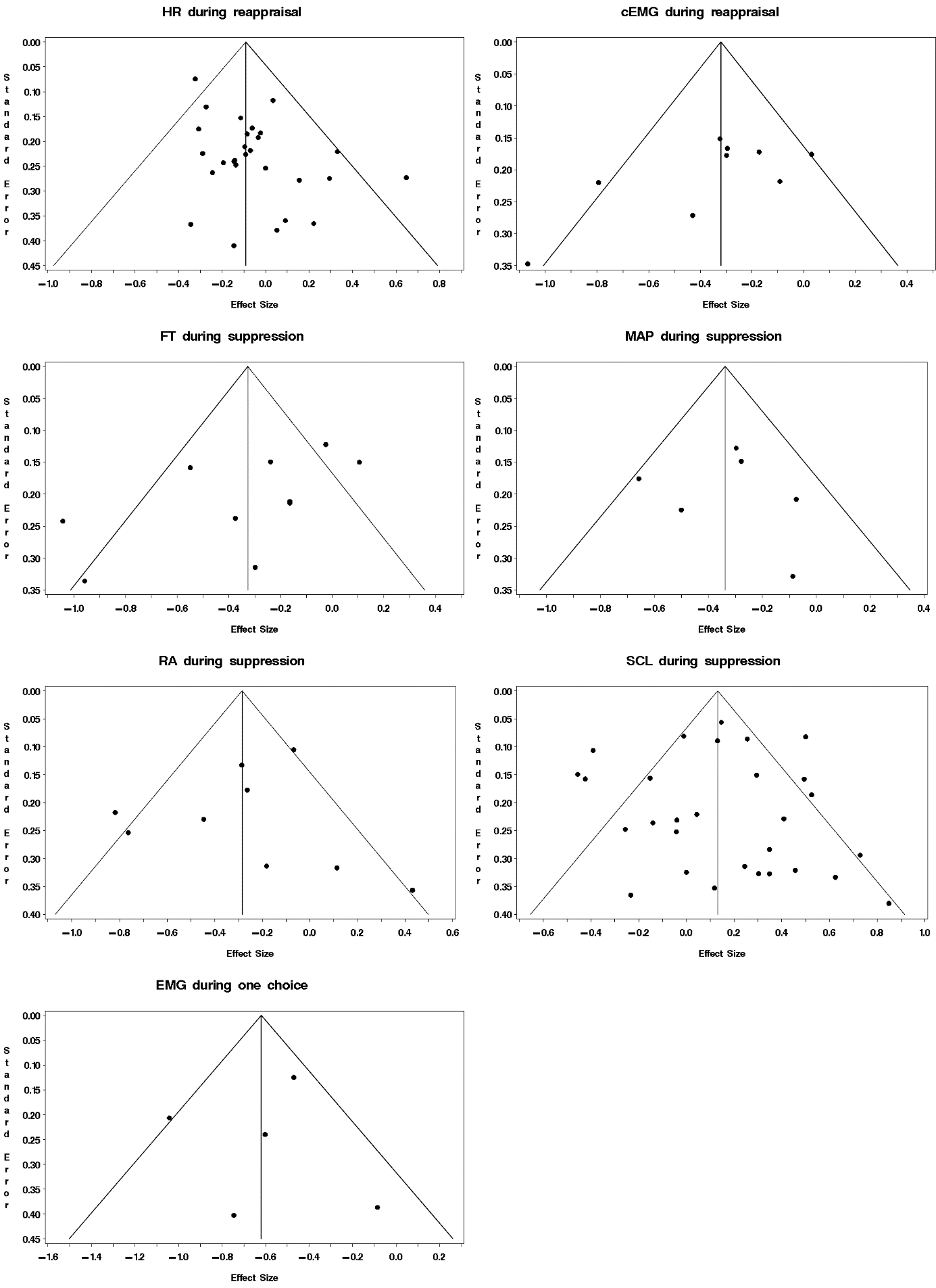

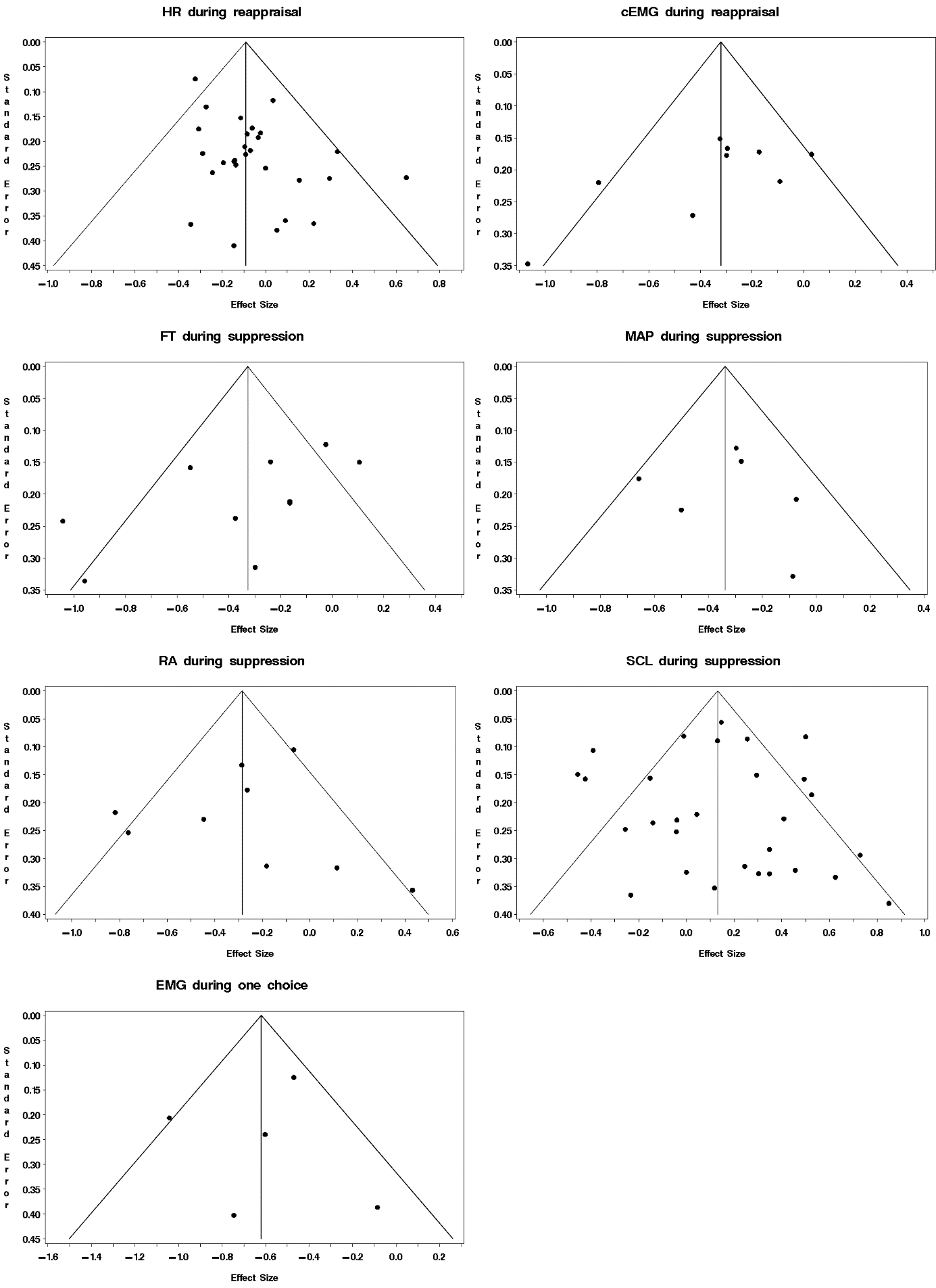


*Figure S24.* Funnel plots with the effect sizes on the horizontal axis and their standard errors on the vertical axis for each significant meta-analysis. Egger’s test revealed that there was significant asymmetry only for the effect of reappraisal on heart rate (HR).

Table S1

*Study characteristics of identified emotion regulation studies inducing positive emotions.*

| **Study** | **Study design** | **Strategy** | **Measure** | **Emotion** | **Control instruction** | **Trial duration** | **Emotion induction** | **N total** | **Percent women** | **Age (mean)** |
| --- | --- | --- | --- | --- | --- | --- | --- | --- | --- | --- |
| Wu, Liang, Wang, Zhao, and Zhou (2016) | W | reappraisal | cEMG | positive | C4 | 6 | I | 75 | 49.33 |  |
| Giuliani, McRae, and Gross (2008) | W | reappraisal | EPPT | positive | C4 | 15 | F | 16 | 100 | 18.8 |
| Giuliani et al. (2008) | W | reappraisal | FPA | positive | C4 | 15 | F | 16 | 100 | 18.8 |
| Giuliani et al. (2008) | W | reappraisal | FPTT | positive | C4 | 15 | F | 16 | 100 | 18.8 |
| Giuliani et al. (2008) | W | reappraisal | FT | positive | C4 | 15 | F | 16 | 100 | 18.8 |
| Giuliani et al. (2008) | W | reappraisal | HR | positive | C4 | 15 | F | 16 | 100 | 18.8 |
| Giuliani et al. (2008) | W | reappraisal | MAP | positive | C4 | 15 | F | 16 | 100 | 18.8 |
| Gruber, Hay, and Gross (2014) | W | reappraisal | nSCR | positive | C1 | 200 | F | 23 | 52.17 | 35.2 |
| Giuliani et al. (2008) | W | reappraisal | RR | positive | C4 | 15 | F | 16 | 100 | 18.8 |
| Giuliani et al. (2008) | W | reappraisal | SCR | positive | C4 | 15 | F | 16 | 100 | 18.8 |
| Gomez, Scholz, and Danuser (2015) | B | reappraisal | SCR | positive | C1 | 10 | I | 81 | 64.2 | 28.2 |
| Wu et al. (2016) | W | reappraisal | zEMG | positive | C4 | 6 | I | 75 | 49.33 |  |
|  |  |  |  |  |  |  |  |  |  |  |
| Baur, Conzelmann, Wieser, and Pauli (2015) | W | own choice | cEMG | positive | C2 | 4 | I | 41 | 78.05 | 21.1 |
| Driscoll, Tranel, and Anderson (2009) | W | own choice | HR | positive | C1 | 8 | I | 10 | 70 | 35.2 |
| Discroll et al., (2009) | W | own choice | SCR | positive | C1 | 8 | I | 10 | 70 | 35.2 |
| Dillon and LaBar (2005), sample 1 | W | own choice | startle | positive | C3 | 12 | I | 48 | 77.08 | 22 |
| Dillon & LaBar (2005), sample 2 | W | own choice | startle | positive | C3 | 12 | I | 48 | 77.08 | 22 |
| Conzelmann, McGregor, and Pauli (2015) | W | own choice | startle | positive | C3 | 8 | I | 31 | 48.39 | 22 |
| Dan-Glauser and Gross (2011) | W | suppression | cEMG | positive | C4 | 8 | I | 37 | 100 | 20.2 |
| Dan-Glauser and Gross (2015) | W | suppression | cEMG | positive | C4 | 8 | I | 37 | 100 | 20.2 |
| Dan-Glauser and Gross (2015) | W | suppression | FPA | positive | C4 | 8 | I | 37 | 100 | 20.2 |

*Table S1 continued*

| **Study** | **Study design** | **Strategy** | **Measure** | **Emotion** | **Control instruction** | **Trial duration** | **Emotion induction** | **N total** | **Percent women** | **Age (mean)** |
| --- | --- | --- | --- | --- | --- | --- | --- | --- | --- | --- |

| Dan-Glauser and Gross (2015) | W | suppression | FPTT | positive | C4 | 8 | I | 37 | 100 | 20.2 |
| --- | --- | --- | --- | --- | --- | --- | --- | --- | --- | --- |
| Dan-Glauser and Gross (2011) | W | suppression | FT | positive | C4 | 8 | I | 37 | 100 | 20.2 |
| Dan-Glauser and Gross (2015) | W | suppression | FT | positive | C4 | 8 | I | 37 | 100 | 20.2 |
| Dan-Glauser and Gross (2011) | W | suppression | HR | positive | C4 | 8 | I | 37 | 100 | 20.2 |
| Dan-Glauser and Gross (2015) | W | suppression | HR | positive | C4 | 8 | I | 37 | 100 | 20.2 |
| Ohira et al. (2006) | W | suppression | HR | positive | C4 | 60 | I | 10 | 100 | 24.2 |
| Gross and Levenson (1997) | B | suppression | HR | amusement | C1 | 210 | F | 180 | 100 |  |
| Gross and Levenson (1997) | B | suppression | HR | amusement | C1 | 210 | F | 180 | 100 |  |
| Dan-Glauser and Gross (2011) | W | suppression | MAP | positive | C4 | 8 | I | 37 | 100 | 20.2 |
| Dan-Glauser and Gross (2015) | W | suppression | MAP | positive | C4 | 8 | I | 37 | 100 | 20.2 |
| Dan-Glauser and Gross (2011) | W | suppression | RA | positive | C4 | 8 | I | 37 | 100 | 20.2 |
| Dan-Glauser and Gross (2015) | W | suppression | RA | positive | C4 | 8 | I | 37 | 100 | 20.2 |
| Dan-Glauser and Gross (2011) | W | suppression | RR | positive | C4 | 8 | I | 37 | 100 | 20.2 |
| Dan-Glauser and Gross (2015) | W | suppression | RR | positive | C4 | 8 | I | 37 | 100 | 20.2 |
| Gross and Levenson (1997) | B | suppression | SCL | amusement | C1 | 210 | F | 180 | 100 |  |
| Gross and Levenson (1997) | B | suppression | SCL | amusement | C1 | 210 | F | 180 | 100 |  |
| Ohira et al. (2006) | W | suppression | SCR | positive | C4 | 60 | I | 10 | 100 | 24.2 |
| Kotwas et al. (2019) | W | suppression | SCR | positive | C4 | 45 | F | 34 | 70.59 | 31.7 |
| Gomez et al. (2015) | B | suppression | SCR | positive | C1 | 10 | I | 81 | 64.2 | 28.2 |
| Dan-Glauser and Gross (2011) | W | suppression | zEMG | positive | C4 | 8 | I | 37 | 100 | 20.2 |
| Dan-Glauser and Gross (2015) | W | suppression | zEMG | positive | C4 | 8 | I | 37 | 100 | 20.2 |

*Note.* cEMG = corrugator electromyography; DBP = diastolic blood pressure; EPTT = ear pulse transit time; FPA = finger pulse amplitude; FPTT = finger pulse transit time; FT = finger temperature; HR = heart rate; HRV = heart rate variability; MAP = mean arterial pressure; PD = pupil dilation; RA = respiration amplitude; RR = respiration rate; SBP = systolic blood pressure; SCL = skin conductance level; SCR = skin conductance response; B = between-subject design study; W = within-subject design study; F = film; I = images; C1 = no instruction given (“view”); C2 = instruction not to regulate; C3 = instruction to maintain target emotion; C4 = instruction to respond naturally; C5 = a

combination of C1-C4; Ntotal = number of participants in the study.

Table S2

*Taxonomy for coding electrodermal measures.*

| **Study** | **Strategy** | **Coded Measure** | **Trial duration (s)** | **Quantification of EDA (Description in paper)** | **Reason for decision** |
| --- | --- | --- | --- | --- | --- |
| Azbel-Jackson, Butler, Ellis, and van Reekum (2015), study 1 | Suppression | SCL | 7 | „Physiological data were baseline corrected by subtracting the signal recorded during the 1 s time window just before stimulus onset from all time points within a 7s time window immediately after picture onset. Data trials that fell more than 4 SDs from the within-subjects mean on a measure-by-measure basis for each participant were eliminated.” | Average |
| Azbel-Jackson et al. (2015), study 2 | Suppression | SCL | 7 |  |  |
| Ben-Naim, Hirschberger, Ein-Dor, and Mikulincer (2013) | Suppression | SCR | 900 | Authors state that skin conductance response and skin conductance level recorded. No specific details about quantification provided. | Max. amplitude/peak |
| Ben-Naim et al. (2013) | Reappraisal | SCR | 900 |  |  |
| Ben-Naim et al. (2013) | Suppression | SCL | 900 |  | Average |
| Ben-Naim et al. (2013) | Reappraisal | SCL | 900 |  |  |
| Butler, Gross, and Barnard (2014) | Suppression | SCL | 570.6 | Authors state that skin conductance level recorded. No specific details about quantification provided. | Author information |
| Butler et al. (2014) | Reappraisal | SCL | 590.8 |  |  |
| Colby, Lanzetta, and Kleck (1977) | Suppression | SCL | 6 | “Skin resistance records were scored at the point of shock onset, 2 sec prior to shock onset and every sec after shock onset up to 6 sec after shock offset. The resistance scores were transformed to mhos of conductance. The phasic skin response for each trial was conducted by subtracting the average of the first two points from the average of the remainder for that particular trial.” | Average |
| Demaree et al. (2006) | Suppression | SCL | 120 | Authors state that skin conductance level recorded. No specific details about quantification provided. | First author provided data on SCL via email. |

*Table S2 continued*

| **Study** | **Strategy** | **Coded Measure** | **Trial duration (s)** | **Quantification of EDA (Description in paper)** | **Reason for decision** |
| --- | --- | --- | --- | --- | --- |

| Driscoll et al. (2009) | Own choice | SCR | 8 | „ Skin conductance response magnitude was scored as the greatest change above 0.02 μS occurring in a 1–4 s time window following picture onset. A log transformation (log [SCR + 1]) was then performed to normalize the SCR data” | Max. amplitude/peak |
| --- | --- | --- | --- | --- | --- |
| Efinger, Thuillard, and Dan-Glauser (2019) | Reappraisal | SCL | 8 | “Skin conductance level was exported as mean values for each trial.” | Average |
| Efinger et al. (2019) | Distraction | SCL | 8 |  |  |
| Fitzpatrick and Kuo (2016) | Distraction | SCL | 10 | "The difference between average SCL during stimuli presentation and average SCL for two seconds prior to the stimuli presentation (during fixation cross presentation) was calculated for each experimental trial and used as the outcome variable." | Average |
| Fuentes‐Sánchez, Jaén, Escrig, Lucas, and Pastor (2019) | Reappraisal | SCR | 8 | “For each trial, the peak response was scored as the maximum EDA value within a 1 to 6 s time window following picture onset, and amplitude was computed as the maximum electrodermal change score with respect to a baseline of 1 s prior to the picture onset.” | Max. amplitude/peak |
| Goldin, Moodie, and Gross (2019) | Reappraisal | SCL | 12 | “Skin conductance level recorded. "We computed mean values for respiration rate, heart rate, and skin conductance for each of the four conditions in the experimental task. We used a lag of 1.5 s, equivalent to one TR in the fMRI time series, to capture autonomic responses." | Average |
| Gomez et al. (2015) | Suppression | SCR | 10 | “To compute SCR change scores were calculated for each picture by subtracting the mean skin conductance of the interval between 1 and 4 sec after picture onset from the peak skin conductance of the interval between 1 and 4 sec after picture onset.” | Max. amplitude/peak |
| Gomez et al. (2015) | Reappraisal | SCR | 10 |  |  |
| Gross (1998) | Suppression | SCL | 64 | Authors state that skin conductance level was recorded. “During the experimental sessions, laboratory software computed second-by-second averages for each of the five physiological measures throughout each baseline, instructional, film, and postfilm period. These second-by-second physiological values were later used to compute scores for each participant representing the averages of the physiological variables for the baseline, instructional, film, and postfilm periods. Change scores for the five measures were computed by subtracting baseline scores from instructional, film, and postfilm periods.” | Average |

*Table S2 continued*

| **Study** | **Strategy** | **Coded Measure** | **Trial duration (s)** | **Quantification of EDA (Description in paper)** | **Reason for decision** |
| --- | --- | --- | --- | --- | --- |

| Gross (1998) | Reappraisal | SCL | 64 | Author state that skin conductance level was recorded. “During the experimental sessions, laboratory software computed second-by-second averages for each of the five physiological measures throughout each baseline, instructional, film, and postfilm period. These seond-by-second physiological values were later used to compute scores for each participant representing the averages of the physiological variables for the baseline, instructional, film, and postfilm periods. Change scores for the five measures were computed by subtracting baseline scores from instructional, film, and postfilm periods.” | Average |
| --- | --- | --- | --- | --- | --- |
| Gross and Levenson (1993), study 1 and 2 | Suppression | SCL | 64 | Authors state that skin conductance level was recorded. “Physiological measures were monitored continuously using an online data acquisition software package developed by our laboratory. This software computed second-by-second averages for each measure.” | Average |
| Gross and Levenson (1997) | Suppression | SCL | 210 | Authors state that skin conductance level was recorded. “Change scores were created by subtracting prefilm period scores from film period scores for each variable.” | Average |
| Hagemann, Levenson, and Gross (2006) | Suppression | SCL | 5 | Authors state that skin conductance level was recorded. "Second-by-second values for each of the physiological measures, except RSA, were then reduced to mean values representing nonoverlapping time slices: (1) 2-min pretrial baseline, (2) 5-s pre-startle period, and (3) 6-s post-startle period (including the startle). Physiological reactivity scores were calculated for each measure by subtracting the pre- trial baseline from the pre- and post-startle periods." | Average |
| Hallam et al. (2015) | Suppression | SCL | 10 | “SCR traces were analysed in Ledalab v.3.2.9 using the Continuous Decomposition Analysis method to distinguish the phasic (driver) information from the underlying tonic sudomotor nerve activity. Raw SCR data were smoothed via convolution with a Hann window to reduce error noise and fitted to a bi-exponential Bateman function. Data were optimised by a conjugated gradient descent algorithm to reduce the error between them and the inbuilt SCR model. These processing steps allowed computation of a stimulus-locked ‘integrated skin conductance response’ (ISCR), a time-integration of the continuous phasic activity for each stimulus. This ISCR therefore represents an unbiased and time-sensitive measure of sympathetic activity in response to each stimulus. For investigating whether implementation intention and goal intention ER strategies may be associated with different skin conductance response, ISCRs from participants in both groups were averaged across epochs, within-subject.” | Integrated signal |
| Hallam et al. (2015) | Reappraisal | SCR | 10 |  |  |

*Table S2 continued*

| **Study** | **Strategy** | **Coded Measure** | **Trial duration (s)** | **Quantification of EDA (Description in paper)** | **Reason for decision** |
| --- | --- | --- | --- | --- | --- |

| Kim and Hamann (2012) | Reappraisal | SCR | 24 | “A skin conductance response was defined as the maximal positive deflection in skin conductance level with its onset occurring within a time window of .5 and 4 sec after each picture presentation. Peak-to-peak amplitude measurement was acquired for each picture. SCR amplitudes less than .03 MicroSiemens or continuously decreasing throughout the window were defined as 0 microSiemens.” | Max. amplitude/peak |
| --- | --- | --- | --- | --- | --- |
| Kinner et al. (2017) | Reappraisal | SCR | 5 | “SCRs were defined as the maximum amplitude within a window of 1-8s after picture onset and calculated as baseline-to-peak amplitude differences of the largest deflection within a time window of 1-8s after picture onset. The baseline was the skin conductance level immediately preceding the inflection point. For each condition individual SCRs were averaged across the 10 trials.” | Max. amplitude/peak |
| Kotwas et al. (2019) | Suppression | SCR | 45 | Only phasic responses of skin conductance were recorded with the amplifier in the AC position. … The mean amplitude of peaks of SCR were measured only during the 45 seconds duration of each film. SCR was obtained by averaging peaks' amplitude for each film. SCR below .01 microSiemens was not considered. | Max. amplitude/peak |
| Kunzmann, Kupperbusch, and Levenson (2005) | Suppression | SCL | 117 | Authors states that skin conductance level was recorded. No specific details about quantification provided. | Author information |
| Leiberg, Eippert, Veit, and Anders (2012) | Reappraisal | SCR | 6 | “EDA responses were determined as the difference between the maximum in the regulation phase (4,000 - 10,000 ms after picture onset) and the maximum in the pre-regulation phase (1,000 - 3,500 ms after picture onset). All trials were included in the analysis, regardless of the size of the response (i.e. reported values are a measure of EDA magnitude).” | Max. amplitude/peak |
| Lohani and Isaacowitz (2014), sample 1 | Suppression | SCL | 300 | For each participant, difference scores for mood, SCL and EMG data were calculated by subtracting the mean activity during baseline period from the respective film period (no-regulation, attentional deployment, positive reappraisal and suppression).For SCL and corrugator, mean values of the respective measures while participants had watched a neutral video were used as baseline activity. | Average |
| Lohani & Isaacowitz (2014), sample 2 | Suppression | SCL | 300 |  |  |
| Lohani & Isaacowitz (2014), sample 1 | Reappraisal | SCL | 300 |  |  |
| Lohani & Isaacowitz (2014), sample 1 | Distraction | SCL | 300 |  |  |

*Table S2 continued*

| **Study** | **Strategy** | **Coded Measure** | **Trial duration (s)** | **Quantification of EDA (Description in paper)** | **Reason for decision** |
| --- | --- | --- | --- | --- | --- |

| Lohani & Isaacowitz (2014), sample 2 | Reappraisal | SCL | 300 |  |  |
| --- | --- | --- | --- | --- | --- |
| Lohani & Isaacowitz (2014), sample 2 | Distraction | SCL | 300 |  |  |
| Morawetz, Bode, Baudewig, Jacobs, and Heekeren (2016a) | Reappraisal | SCR | 8 | “Values for phasic SCRs were extracted as the difference between a local minimum and the succeeding local maximum within the response window“ | Max. amplitude/peak |
| Morawetz et al. (2016b) | Reappraisal | SCR | 8 | “Skin conductance responses were defined as a deflection of at least .01 microSiemens occurring 1-8 s after stimulus onset. Only runs including more than 10% SCRs exceeding the above criterion were used for analysis. Values for phasic SCRs were extracted as the difference between a local minimum and the succeeding local maximum within the response window.” | Max. amplitude/peak |
| Morawetz, Bode, Baudewig, and Heekeren (2017) | Reappraisal | SCR | 8 | “Values for phasic SCRs were extracted as the difference between a local minimum and the succeeding local maximum within the re- sponse window.” | Max. amplitude/peak |
| Ohira et al. (2006) | Suppression | SCR | 60 | “The amplitude of the maximum peak of SCR was measured in every 10s-time window during the presentation of each stimulus. Mean scores of SCRs during each block were analyzed statistically.” | Max. amplitude/peak |
| Opitz, Lee, Gross, and Urry (2014), sample 1 | Reappraisal | SCL | 8 | EDA level recorded. "For the continuous peripheral physiological measures, we summarized raw activity for two periods of interest in each trial, pre- instruction activity (reactivity period; mean of activity occurring 4 s after picture onset) and post-instruction activity (regulation period; mean of activity occurring 8 s after both ER manipulations)." | Average |
| Opitz et al. (2014), sample 2 | Reappraisal | SCL | 8 |  |  |
| Ortner (2015) | Reappraisal | SCR | 8 | “Skin conductance levels were collected throughout the task, and SCRs to each picture were calculated by subtracting the mean skin conductance response for the 1,000 ms prior to picture onset from the maximum during the 8,000 ms of picture presentation.” | Max. amplitude/peak |
| Plieger et al. (2017) | Reappraisal | SCL | 4.5 | “After smoothing the data, we conducted a continuous decomposition analysis (CDA) to separate the phasic parts from the tonic parts. Hence, the skin conductance response (SCR) reported here is adjusted by tonic activity. We measured phasic activity for 5 s starting with the onset of each stimulus. Phasic activity was averaged across all blocks of negative stimuli and across all blocks of neutral stimuli.” | Average |

*Table S2 continued*

| **Study** | **Strategy** | **Coded Measure** | **Trial duration (s)** | **Quantification of EDA (Description in paper)** | **Reason for decision** |
| --- | --- | --- | --- | --- | --- |

| Richards and Gross (1999) | Suppression | SCL | 84 | “Responses were digitized using custom software, which also computed second-by-second period averages for each of the five measures. In addition, change scores were calculated for each physiological measure by subtracting the baseline average from each slide-viewing period average .” | Average |
| --- | --- | --- | --- | --- | --- |
| Roberts, Levenson, and Gross (2008), sample 1 | Suppression | SCL | 62 | For the remaining six measures (systolic and diastolic blood pressure, cardiac interbeat interval, RSA, skin conductance level, and general somatic activity), separate change scores were computed by subtracting mean response during the two-minute pre-film baseline from mean response during the film period | Average |
| Roberts et al. (2008), sample 2 | Suppression | SCL | 62 |  |  |
| Roberts et al. (2008), sample 3 | Suppression | SCL | 62 |  |  |
| Roberts et al. (2008), sample 4 | Suppression | SCL | 62 |  |  |
| Robinson and Demaree (2009) | Suppression | SCL | 120 | Author state that (galvanic) skin conductance level was recorded. "Skin conductance data were also collected via the use of Biopac TSD203 transducers with Biopac Skin Conductance Electrode Paste placed at the non-dominant middle and fourth fingers. Data were amplified using Biopac’s GSR100C amplifier using a gain of 10 lmhos and a low-pass filter of 10 Hz. Mindware’s EDA 2.1 computer program identified all galvanic skin responses (SCR), as defined as a .05 lS increase in skin conductance." Report Galvanic skin conductance level in results section. No further specification about skin conductance responses. | Author information |
| Rohrmann, Hopp, Schienle, and Hodapp (2009), sample 1 | Suppression | SCL | 60 | “Because of the high interindividual variability of the skin conductance level a range correction according to Schandry (1998) was performed [corrected EDA-value = EDA-value minus minimal EDA-value/maximum EDA- value minus minimal EDA-value] in order to obtain values independent from the individual absolute level. Heart rate (bpm) as well as skin conductance (mS) values were averaged across the period of the neutral and the amputation film.” | Average |
| Rohrmann et al. (2009), sample 2 | Suppression | SCL | 60 |  |  |
| Rohrmann et al. (2009), sample 1 | Reappraisal | SCL | 60 |  |  |

*Table S2 continued*

| **Study** | **Strategy** | **Coded Measure** | **Trial duration (s)** | **Quantification of EDA (Description in paper)** | **Reason for decision** |
| --- | --- | --- | --- | --- | --- |

| Rohrmann et al. (2009), sample 2 | Reappraisal | SCL | 60 |  |  |
| --- | --- | --- | --- | --- | --- |
| Roth et al. (2014) | Suppression | SCL | 197 | “The second-by-second SCL values were averaged for the two epochs of the baseline and the fear-eliciting film in each session. A difference score was calculated by subtracting the baseline mean SCL from the value obtained during the fear-eliciting film.” | Average |
| Roth et al., (2014) | Distraction | SCL | 197 |  |  |
| Sheppes, Catran, and Meiran (2009) | Reappraisal | SCL | 190 | Authors state that skin conductance level was recorded. "For all measures, we applied the mean change from the pre-instruction baseline score, using the AcqKnowledge software (Biopac Systems, Goleta, CA)." | Average |
| Sheppes et al. (2009) | Distraction | SCL | 190 |  |  |
| Shermohammed et al. (2017) | Reappraisal | SCR | 8 | “The amplitude of the skin conductance response for each trial was calculated using the maximum change from base to peak in the .5-4.5 sec after picture onset. Amplitudes below .02 Microsec were scored as 0.” | Max. amplitude/peak |
| Shiota and Levenson (2009), sample 1 | Suppression | SCL | 180 | Author state that skin conductance level was recorded. No specific details about quantification provided. | Author information |
| Shiota and Levenson (2009), sample 2 | Reappraisal | SCL | 180 |  |  |
| Shiota and Levenson (2009), sample 3 | Reappraisal | SCL | 180 |  |  |
| Shiota and Levenson (2009), sample 4 | Suppression | SCL | 180 |  |  |
| Shiota and Levenson (2009), sample 5 | Reappraisal | SCL | 180 |  |  |
| Shiota and Levenson (2009), sample 6 | Reappraisal | SCL | 180 |  |  |
| Shiota and Levenson (2009), sample 7 | Suppression | SCL | 180 |  |  |
| Shiota and Levenson (2009), sample 8 | Reappraisal | SCL | 180 |  |  |
| Shiota and Levenson (2009), sample 9 | Reappraisal | SCL | 180 |  |  |

*Table S2 continued*

| **Study** | **Strategy** | **Coded Measure** | **Trial duration (s)** | **Quantification of EDA (Description in paper)** | **Reason for decision** |
| --- | --- | --- | --- | --- | --- |

| Soto, Lee, and Roberts (2016) | Suppression | SCL | 58 | “Mean reactivity levels were calculated for both IBI and SCL by subtracting the mean of the 1-minute prefilm baseline from mean responses during each film.” | Average |
| --- | --- | --- | --- | --- | --- |
| Stiller, Kattner, Gunzenhauser, and Schmitz (2019) | Suppression | SCL | 165 | No specific information. Average skin conductance reported. | Average |
| Stiller et al. (2019) | Reappraisal | SCL | 165 |  |  |
| Svaldi, Caffier, and Tuschen-Caffier (2010) | Suppression | SCL | 211 | Authors state that skin conductance level was recorded. No specific details about quantification provided. | Author information |
| Svaldi et al. (2010) | Reappraisal | SCL | 125 |  |  |
| Urry (2009) | Reappraisal | SCR | 8 | “Reappraisal- related change was thus captured by subtracting the baseline signal recorded during the 100-ms (EDA, HR) or 250-ms (EMG) time bin just before instruction delivery from all time points within an 8-s window immediately after this baseline period. During anticipatory trials the instruction was delivered 2 s before picture onset, and during online trials the instruction was delivered 4 s after picture onset. After baseline correction, the 8-s reappraisal window of interest was subdivided into early (first 4 s) and late (last 4 s) periods to enable detection of changes in the effect of reappraisal goal over time. The data were then aggregated across trials within each cell (described by reappraisal goal, timing, and period) for each participant. The summary statistic (mean for corrugator EMG and HR, maximum for EDA) was then tested with a multivariate GLM to assess the effects of reappraisal timing (anticipatory vs. online), reappraisal goal (increase, maintain, decrease), and time period (early, late).” | Max. amplitude/peak |
| Urry (2010) | Reappraisal | SCL | 4 | “Reappraisal-related change was captured by subtracting baseline signal recorded during the 100-ms (EDA, HR) or 250-ms (EMG) time bin just prior to instruction delivery from all subsequent time bins within a 6-second window immediately following this baseline period. To account for delay in EDA response, only time bins occurring 2 seconds after instruction delivery were included. Following baseline correction, the data were then averaged across time bins and trials within each cell (described by reappraisal goal and gaze direction) for each participant.” | Average |

*Table S2 continued*

| **Study** | **Strategy** | **Coded Measure** | **Trial duration (s)** | **Quantification of EDA (Description in paper)** | **Reason for decision** |
| --- | --- | --- | --- | --- | --- |

| Urry, van Reekum, Johnstone, and Davidson (2009) | Reappraisal | SCL | 8 | “The data were downsampled to 20 Hz offline, averaged across trials within each condition in 1-s time bins, and linearly detrended for each participant by trial block (24 trials for the regulation task). Regulation-related change in EDA was captured by subtracting EDA during the 4-s picture period from each of eight 1-s time bins following delivery of the reappraisal instruction. An area-under-the-curve index of EDA was computed by summing across all time points within each of three time intervals, early, middle, and late, as described below for the pupil diameter measure.” | Area under the curve |
| --- | --- | --- | --- | --- | --- |
| Wolgast, Lundh, and Viborg (2011) | Reappraisal | SCL | 153 | “Average values for SCL were calculated for each film-clip using the software EDFBrowser and SPSS.” | Average |
| Wu et al. (2016), study 2 | Reappraisal | SCL | 180 | Biotrace+ software (Mind Media B.V., Netherlands) supplied with the NeXus-10 was applied to data reduction, artifact control, and computation of average SCL scores for each participant for each 3-min relaxation period and film clip. |  |
| Yuan, Liu, Ding, and Yang (2014) | Suppression | SCL | 1800 | No specific information about quantification provided. “Average values of SCR were computed for each phase of interest (e.g. rest, task, recovery) using the BioTrace+ software.” | Average |

*Note.* SCL = skin conductance level; SCR = skin conductance response; Text in quotations marks are passages directly quoted from the paper.

**References**

Azbel-Jackson, L., Butler, L. T., Ellis, J. A., & van Reekum, C. M. (2015). Stay calm! Regulating emotional responses by implementation intentions: Assessing the impact on physiological and subjective arousal. *Cognition and Emotion*, 1-15. doi:10.1080/02699931.2015.1049515

Baur, R., Conzelmann, A., Wieser, M. J., & Pauli, P. (2015). Spontaneous emotion regulation: Differential effects on evoked brain potentials and facial muscle activity. *International Journal of Psychophysiology, 96*(1), 38-48. doi:10.1016/j.ijpsycho.2015.02.022

Ben-Naim, S., Hirschberger, G., Ein-Dor, T., & Mikulincer, M. (2013). An experimental study of emotion regulation during relationship conflict interactions: The moderating role of attachment orientations. *Emotion, 13*(3), 506-519. doi:10.1037/a0031473

Boucsein, W., Fowles, D. C., Grimnes, S., Ben-Shakhar, G., roth, W. T., Dawson, M. E., & Filion, D. L. (2012). Publication recommendations for electrodermal measurements. *Psychophysiology, 49*(8), 1017-1034. doi:10.1111/j.1469-8986.2012.01384.x

Butler, E. A., Gross, J. J., & Barnard, K. (2014). Testing the effects of suppression and reappraisal on emotional concordance using a multivariate multilevel model. *Biol Psychol, 98*, 6-18. doi:10.1016/j.biopsycho.2013.09.003

Colby, C. Z., Lanzetta, J. T., & Kleck, R. E. (1977). Effects of the Expression of Pain on Autonomic and Pain Tolerance Responses to Subject‐Controlled Pain. *Psychophysiology, 14*(6), 537-540. doi:10.1111/j.1469-8986.1977.tb01194.x

Conzelmann, A., McGregor, V., & Pauli, P. (2015). Emotion regulation of the affect-modulated startle reflex during different picture categories. *Psychophysiology, 52*(9), 1257-1262. doi:10.1111/psyp.12450

Dan-Glauser, E. S., & Gross, J. J. (2011). The temporal dynamics of two response-focused forms of emotion regulation: experiential, expressive, and autonomic consequences. *Psychophysiology, 48*(9), 1309-1322. doi:10.1111/j.1469-8986.2011.01191.x

Dan-Glauser, E. S., & Gross, J. J. (2015). The temporal dynamics of emotional acceptance: Experience, expression, and physiology. *Biological Psychology, 108*, 1-12. doi:10.1016/j.biopsycho.2015.03.005

Demaree, H. A., Schmeichel, B. J., Robinson, J. L., Pu, J., Everhart, D. E., & Berntson, G. G. (2006). Up- and down-regulating facial disgust: affective, vagal, sympathetic, and respiratory consequences. *Biological Psychology, 71*(1), 90-99. doi:10.1016/j.biopsycho.2005.02.006

Dillon, D. G., & LaBar, K. S. (2005). Startle modulation during conscious emotion regulation is arousal-dependent. *Behavioral neuroscience, 119*(4), 1118-1124. doi:10.1037/0735-7044.119.4.1118

Driscoll, D., Tranel, D., & Anderson, S. W. (2009). The effects of voluntary regulation of positive and negative emotion on psychophysiological responsiveness. *International Journal of Psychophysiology, 72*(1), 61-66. doi:10.1016/j.ijpsycho.2008.03.012

Efinger, L., Thuillard, S., & Dan-Glauser, E. (2019). Distraction and reappraisal efficiency on immediate negative emotional responses: role of trait anxiety. *Anxiety, Stress, & Coping, 32*(4), 412-427. doi:10.1080/10615806.2019.1597859

Fitzpatrick, S., & Kuo, J. R. (2016). The impact of stimulus arousal level on emotion regulation effectiveness in borderline personality disorder. *Psychiatry Research, 241*, 242-248. doi:10.1016/j.psychres.2016.05.004

Fuentes‐Sánchez, N., Jaén, I., Escrig, M. A., Lucas, I., & Pastor, M. C. (2019). Cognitive reappraisal during unpleasant picture processing: Subjective self‐report and peripheral physiology. *Psychophysiology*, e13372. doi:10.1111/psyp.13372

Giuliani, N. R., McRae, K., & Gross, J. J. (2008). The up- and down-regulation of amusement: experiential, behavioral, and autonomic consequences. *Emotion, 8*(5), 714-719. doi:10.1037/a0013236

Goldin, P. R., Moodie, C. A., & Gross, J. J. (2019). Acceptance versus reappraisal: Behavioral, autonomic, and neural effects. *Cognitive, Affective, & Behavioral Neuroscience, 19*, 927-944. doi:10.3758/s13415-019-00690-7

Gomez, P., Scholz, U., & Danuser, B. (2015). The Down-Regulation of Disgust by Implementation Intentions: Experiential and Physiological Concomitants. *Applied Psychophysiology and Biofeedback, 40*(2), 95-106. doi:10.1007/s10484-015-9280-2

Gross, J. J. (1998). Antecedent-and response-focused emotion regulation: divergent consequences for experience, expression, and physiology. *Journal of Personality and Social Psychology, 74*(1), 224-237. doi:10.1037/0022-3514.74.1.224

Gross, J. J., & Levenson, R. W. (1993). Emotional suppression: physiology, self-report, and expressive behavior. *Journal of Personality and Social Psychology, 64*(6), 970-986. doi:10.1037//0022-3514.64.6.970

Gross, J. J., & Levenson, R. W. (1997). Hiding feelings: The acute effects of inhibiting negative and positive emotion. *Journal of abnormal psychology, 106*(1), 95-103. doi:10.1037/0021-843x.106.1.95

Gruber, J., Hay, A. C., & Gross, J. J. (2014). Rethinking emotion: Cognitive reappraisal is an effective positive and negative emotion regulation strategy in bipolar disorder. *Emotion, 14*(2), 388-396. doi:10.1037/a0035249

Hagemann, T., Levenson, R. W., & Gross, J. J. (2006). Expressive suppression during an acoustic startle. *Psychophysiology, 43*(1), 104-112. doi:10.1111/j.1469-8986.2006.00382.x

Hallam, G. P., Webb, T. L., Sheeran, P., Miles, E., Wilkinson, I. D., Hunter, M. D., . . . Farrow, T. F. D. (2015). The Neural Correlates of Emotion Regulation by Implementation Intentions. *PLoS ONE, 10*(3). doi:10.1371/journal.pone.0119500

Kim, S. H., & Hamann, S. (2012). The effect of cognitive reappraisal on physiological reactivity and emotional memory. *International Journal of Psychophysiology, 83*(3), 348-356. doi:0.1016/j.ijpsycho.2011.12.001

Kinner, V. L., Kuchinke, L., Dierolf, A. M., Merz, C. J., Otto, T., & Wolf, O. T. (2017). What our eyes tell us about feelings: Tracking pupillary responses during emotion regulation processes. *Psychophysiology, 54*(4), 508-518. doi:10.1111/psyp.12816

Kotwas, I., Micoulaud-Franchi, J. A., Khalfa, S., McGonigal, A., Bastien-Toniazzo, M., & Bartolomei, F. (2019). Subjective and physiological response to emotions in temporal lobe epilepsy and psychogenic non-epileptic seizures. *Journal of Affective Disorders, 244*, 46-53. doi:10.1016/j.jad.2018.10.004

Kunzmann, U., Kupperbusch, C. S., & Levenson, R. W. (2005). Behavioral inhibition and amplification during emotional arousal: a comparison of two age groups. *Psychol Aging, 20*(1), 144-158. doi:10.1037/0882-7974.20.1.144

Leiberg, S., Eippert, F., Veit, R., & Anders, S. (2012). Intentional social distance regulation alters affective responses towards victims of violence: an FMRI study. *Hum Brain Mapp, 33*(10), 2464-2476. doi:10.1002/hbm.21376

Lohani, M., & Isaacowitz, D. M. (2014). Age differences in managing response to sadness elicitors using attentional deployment, positive reappraisal and suppression. *Cognition & emotion, 28*(4), 678-697 doi:10.1080/02699931.2013.853648

Morawetz, C., Bode, S., Baudewig, J., & Heekeren, H. R. (2017). Effective amygdala-prefrontal connectivity predicts individual differences in successful emotion regulation. *Soc Cogn Affect Neurosci, 12*(4), 569-585. doi:10.1093/scan/nsw169

Morawetz, C., Bode, S., Baudewig, J., Jacobs, A. M., & Heekeren, H. R. (2016a). Neural representation of emotion regulation goals. *Human brain mapping, 37*(2), 600-620. doi:10.1002/hbm.23053

Morawetz, C., Kellermann, T., Kogler, L., Radke, S., Blechert, J., & Derntl, B. (2016b). Intrinsic functional connectivity underlying successful emotion regulation of angry faces. *Social Cognitive and Affective Neuroscience, 11*(12), 1980-1991. doi:10.1093/scan/nsw107

Ohira, H., Nomura, M., Ichikawa, N., Isowa, T., Iidaka, T., Sato, A., . . . Yamada, J. (2006). Association of neural and physiological responses during voluntary emotion suppression. *NeuroImage, 29*(3), 721-733. doi:10.1016/j.neuroimage.2005.08.047

Opitz, P. C., Lee, I. A., Gross, J. J., & Urry, H. L. (2014). Fluid cognitive ability is a resource for successful emotion regulation in older and younger adults. *Frontiers in psychology, 5*, 609. doi:10.3389/fpsyg.2014.00609

Ortner, C. N. M. (2015). Divergent effects of reappraisal and labeling internal affective feelings on subjective emotional experience. *Motivation and Emotion, 39*(4), 563-570. doi:10.1007/s11031-015-9473-2

Plieger, T., Melchers, M., Vetterlein, A., Görtz, J., Kuhn, S., Ruppel, M., & Reuter, M. (2017). The serotonin transporter polymorphism (5-HTTLPR) and coping strategies influence successful emotion regulation in an acute stress situation: Physiological evidence. *International Journal of Psychophysiology, 114*, 31-37. doi:10.1016/j.ijpsycho.2017.02.006

Richards, J. M., & Gross, J. J. (1999). Composure at any cost? The cognitive consequences of emotion suppression. *Personality and Social Psychology Bulletin, 25*(8), 1033-1044. doi:10.1177/01461672992511010

Roberts, N. A., Levenson, R. W., & Gross, J. J. (2008). Cardiovascular costs of emotion suppression cross ethnic lines. *International Journal of Psychophysiology, 70*(1), 82-87. doi:10.1016/j.ijpsycho.2008.06.003

Robinson, J. L., & Demaree, H. A. (2009). Experiencing and regulating sadness: Physiological and cognitive effects. *Brain and cognition, 70*(1), 13-20. doi:10.1016/j.bandc.2008.06.007

Rohrmann, S., Hopp, H., Schienle, A., & Hodapp, V. (2009). Emotion regulation, disgust sensitivity, and psychophysiological responses to a disgust-inducing film. *Anxiety, Stress & Coping: An International Journal, 22*(2), 215-236. doi:10.1080/10615800802016591

Roth, G., Benita, M., Amrani, C., Shachar, B. H., Asoulin, H., Moed, A., . . . Kanat-Maymon, Y. (2014). Integration of negative emotional experience versus suppression: addressing the question of adaptive functioning. *Emotion, 14*(5), 908-919. doi:10.1037/a0037051

Sheppes, G., Catran, E., & Meiran, N. (2009). Reappraisal (but not distraction) is going to make you sweat: Physiological evidence for self-control effort. *International Journal of Psychophysiology, 71*(2), 91-96. doi:10.1016/j.ijpsycho.2008.06.006

Shermohammed, M., Mehta, P. H., Zhang, J., Brandes, C. M., Chang, L. J., & Somerville, L. H. (2017). Does psychosocial stress impact cognitive reappraisal? Behavioral and neural evidence. *Journal of Cognitive Neuroscience, 29*(11), 1803-1816. doi:10.1162/jocn_a_01157

Shiota, M. N., & Levenson, R. W. (2009). Effects of aging on experimentally instructed detached reappraisal, positive reappraisal, and emotional behavior suppression. *Psychol Aging, 24*(4), 890-900. doi:10.1037/a0017896

Soto, J. A., Lee, E. A., & Roberts, N. A. (2016). Convergence in feeling, divergence in physiology: How culture influences the consequences of disgust suppression and amplification among European Americans and Asian Americans. *Psychophysiology, 53*(1), 41-51. doi:10.1111/psyp.12579

Stiller, A.-K., Kattner, M. F., Gunzenhauser, C., & Schmitz, B. (2019). The effect of positive reappraisal on the availability of self-control resources and self-regulated learning. *Educational Psychology, 39*(1), 86-111. doi:10.1080/01443410.2018.1524851

Svaldi, J., Caffier, D., & Tuschen-Caffier, B. (2010). Emotion suppression but not reappraisal increases desire to binge in women with binge eating disorder. *Psychother Psychosom, 79*(3), 188-190. doi:10.1159/000296138

Urry, H. L. (2009). Using Reappraisal To Regulate Unpleasant Emotional Episodes: Goals and Timing Matter. *Emotion, 9*(6), 782-797. doi:10.1037/a0017109

Urry, H. L. (2010). Seeing, Thinking, and Feeling: Emotion-Regulating Effects of Gaze-Directed Cognitive Reappraisal. *Emotion, 10*(1), 125-135. doi:10.1037/a0017434

Urry, H. L., van Reekum, C. M., Johnstone, T., & Davidson, R. J. (2009). Individual differences in some (but not all) medial prefrontal regions reflect cognitive demand while regulating unpleasant emotion. *NeuroImage, 47*(3), 852-863. doi:10.1016/j.neuroimage.2009.05.069

Wolgast, M., Lundh, L.-G., & Viborg, G. (2011). Cognitive reappraisal and acceptance: An experimental comparison of two emotion regulation strategies. *Behaviour research and therapy, 49*(12), 858-866. doi:10.1016/j.brat.2011.09.011

Wu, M., Liang, Y., Wang, Q., Zhao, Y., & Zhou, R. (2016). Emotion dysregulation of women with premenstrual syndrome. *Sci Rep, 6*, 38501. doi:10.1038/srep38501

Yuan, J., Liu, Y., Ding, N., & Yang, J. (2014). The regulation of induced depression during a frustrating situation: Benefits of expressive suppression in Chinese individuals. *PLoS ONE, 9*(5), e97420. doi:10.1371/journal.pone.0097420
